# mHealth Intervention to Promote Nonexercise Physical Activity in Patients With Type 2 Diabetes: Secondary Analysis and Implementation Study

**DOI:** 10.2196/80304

**Published:** 2026-03-19

**Authors:** Minna Aittasalo, Kari Tokola, Henri Vähä-Ypyä, Pauliina Husu, Ari Mänttäri, Tuula Martiskainen, Tiina Laatikainen, Harri Sievänen

**Affiliations:** 1 The UKK Institute for Health Promotion Research, Tampere, Finland; 2 Laurea University of Applied Sciences, Ratatie 22, Vantaa, 01300, Finland, 358 50 346 5908; 3 Wellbeing Services County of North Karelia (Siun Sote), Joensuu, Finland; 4 Institute of Public Health and Clinical Nutrition, University of Eastern Finland, Kuopio, Finland

**Keywords:** accelerometer, 24/7 physical activity, smartphone app, feasibility, physical activity counseling

## Abstract

**Background:**

Physical activity (PA) has an important role in the prevention and treatment of type 2 diabetes (T2D). Interventions with mobile-based technology (mobile health [mHealth]) seem promising in PA promotion, but their behavioral framework is often vague, and the implementation is seldom reported.

**Objective:**

This paper examines perceived behavior change needs and implementation of an mHealth approach in increasing nonexercise PA in patients with T2D.

**Methods:**

A 3-arm mHealth intervention was conducted in primary care. Information on perceived behavior change needs was collected with a modified capability, opportunity, motivation—behavior (COM-B) questionnaire before the intervention from a separate sample of patients with T2D (n=25) and at the intervention baseline (n=119). Implementation evaluation focused on the fidelity and acceptability of the main arm of the intervention (n=39), which included 24-hour accelerometer use, a smartphone app with personal feedback, a PA leaflet, a YouTube video on walking, and individual counseling with 3 face-to-face sessions and 4 telephone contacts. Data on fidelity were accumulated during the intervention through counseling cards and cloud computing. Data on acceptability were collected with a questionnaire at the end of the intervention (Likert scale from 1 to 5). Data analysis was mainly descriptive.

**Results:**

The participants’ responses revealed 3 items in capability and 2 in motivation, which stood out as perceived behavior change needs. Moreover, the main intervention arm showed good fidelity (eg, face-to-face sessions: 112/117, 96% and telephone contacts completed: 145/156, 93%; mean weekly accelerometer use 54%; ranging from 80% to 17% during the intervention) and acceptability (mean score ranging from 3.8 to 4.8), although some challenges were also experienced, especially in cloud-computed feedback and accelerometer-app use.

**Conclusions:**

The findings on behavior change needs call for additional research since no comparable studies were found. In addition, the explanatory value of the COM-B model and the psychometric properties of the COM-B questionnaire deserve further attention. The main intervention arm seemed applicable to clinical practice. However, the challenges discovered underscore the importance of pretesting technology-based approaches in patients with T2D.

## Introduction

Type 2 diabetes (T2D) is a global health problem, and its prevalence is steadily increasing [1,2]. In Finland, T2D has become a major public health threat, increasing in parallel with obesity [3,4]. The management of T2D, cardiovascular diseases, and their risk factors accounts for a substantial part of the burden in primary health care [2].

Physical activity (PA) is one of the cornerstones of the T2D treatment [5,6]. The current guideline for patients with T2D is to spread at least 150 minutes of moderate-to-vigorous intensity physical activity (MVPA) on most days of the week, with no more than 2 days between the activity bouts [7]. Unfortunately, the recommendation seems not achievable to many patients with T2D, especially among women and those who are old and have a high BMI [8].

Focusing on the nonexercise part of the PA continuum, such as replacing sedentary behavior with light-intensity physical activity (LIPA), may offer a feasible way for individuals with clinical conditions to start changing their PA behavior [9-11]. A recent systematic review with meta-analysis shows that compared with whole-day sitting without interruptions, frequent short bouts of LIPA confer favorable acute effects on postprandial glucose and insulin levels in people with metabolic impairments [9]. In many studies, the beneficial metabolic effects have been sustained until the next day [12-14]. Consequently, reducing sedentary time by interrupting prolonged sitting every 30 minutes with short bouts of LIPA has also been included in PA recommendations for patients with T2D [6,7].

The findings on the long-term benefits of breaking up sedentary time [7] or time spent in LIPA for patients with T2D are less clear [9,15]. It seems that in terms of a single-time unit, MVPA still has clear health advantages over LIPA [9,15,16], meaning that to gain the health benefits of MVPA, LIPA should last much longer. However, in a situation where there is very little or no MVPA, as it is often with many people with clinical conditions [17], the time spent in LIPA may also play a substantial health-enhancing role [11,14,18]. This is supported by a meta-analysis showing that exercise of any intensity lasting 12 weeks or more can produce favorable effects on glycemic outcomes in patients with T2D [19]. Similar findings have been discovered for walking as a specific mode of PA [20].

Mobile health (mHealth) interventions offer a promising approach to promoting PA in the general population [21,22]. Encouraging results [23,24] and protocols of new studies [25-27] in patients with T2D have been reported. However, behavioral theories are underused in designing these interventions [28], although their use has been shown to associate with improved intervention outcomes [29,30]. The use of theoretical frameworks has also been unclearly reported [31] and inappropriately linked to the expected mechanisms of behavior change and intervention constructs [32]. Making inaccurate assumptions of what needs to change may lead to failures in behavior change interventions [33].

An intervention can also fail due to incorrect or poor delivery, which emphasizes the evaluation of implementation along with effectiveness [34]. Moreover, information on implementation is critical for translating the interventions into real-world settings [35]. Despite its importance, implementation is inconsistently and insufficiently reported in interventions promoting PA in patients with T2D [36]. As part of the implementation, a notable gap exists in research concerning the perceptions of patients with T2D about mHealth technology [37-39]. Previous studies indicate large variability in the usability of mHealth apps among patients with T2D [29,40] and that satisfaction can improve the adoption of technology-assisted interventions [41].

The purpose of this study is to examine (1) the perceived behavior change needs of patients with T2D for increasing daily walking and other nonexercise PA and (2) the implementation of an mHealth approach to increase patients’ daily walking and other nonexercise PA. These were the secondary aims of the clinical intervention trial. The main aim was to examine the effectiveness of the trial in increasing the daily steps of the patients, which was, however, compromised due to the outbreak of the COVID-19 pandemic in 2020. Thus, only the results concerning the secondary aims are reported here.

## Methods

### Intervention

The 6-month mHealth trial called “MySteps” was implemented within the primary care of the joint municipal authority for North Karelia social and health services (Siun Sote) in Eastern Finland during 2019 to 2020. By design, the trial was a randomized controlled trial and comprised 2 intervention arms and 1 comparison arm.

#### Providers and Participants

Two physiotherapists recruited the patients with T2D for the MySteps trial from the primary care units of the Siun Sote area and through a local newspaper. Inclusion criteria for participation were age 20-69 years, BMI of <40 kg/m^2^, not meeting the current PA recommendation for health, and the ability to conduct a 6-minute walk test [42]. Exclusion criteria for participation were any problem that limited the ability to walk without an aid, any health problem that hindered participation in PA, and inability or unwillingness to use a smartphone or wear an accelerometer.

#### Measurements

A face-to-face session for the baseline measurements was arranged for each participant individually. The measurements included 7-day 24-hour accelerometer use, sleep diary, 6-minute walk test, and blood samples, which are not reported here. As part of the baseline measurements, the participants completed a questionnaire, which, among other things, included questions on intensity-specific PA and perceived behavior change needs for increasing daily walking and other nonexercise PA.

#### Delivery

After the baseline measurements, the participants were randomized into three arms: (1) self-monitoring with counseling (SMC), (2) self-monitoring only, and (3) usual care. SMC was considered the primary intervention arm of the study and is the focus of the implementation evaluation of this paper.

**Figure 1.**
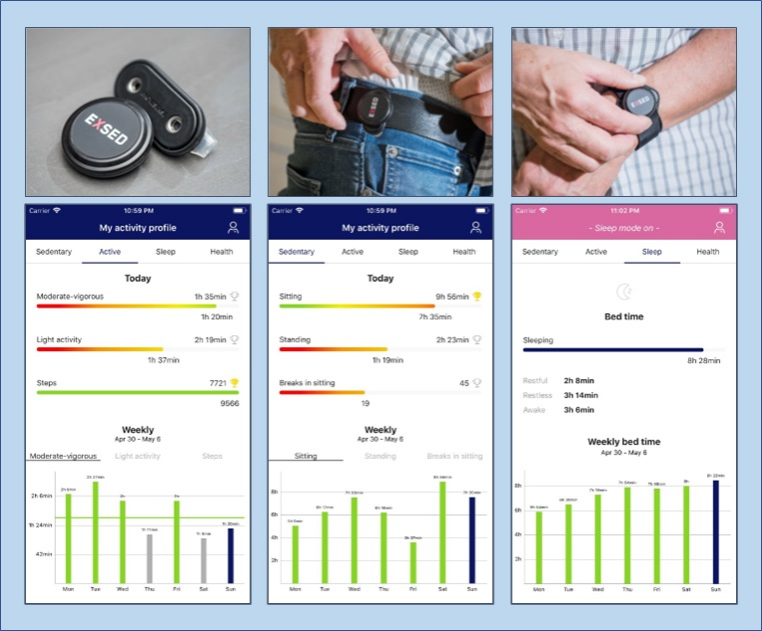
Accelerometer used in self-monitoring and examples of cloud-computed feedback views in the smartphone app (from left to right: physical activity, sedentary behavior, and sleep). The accelerometer was carried on the right hip during waking hours, replaced to the wrist for the time in bed, and instructed to be removed for any water exposure.

SMC was built around an mHealth system, which included an accelerometer (MoveSense, Suunto Ltd) and a smartphone app (ExSed 2, UKK Terveyspalvelut Oy) for 26 weeks. The accelerometer was connected to the app via Bluetooth. The app provided cloud-computed personal data and feedback on accelerometer-measured PA, sedentary behavior, and time in bed (a proxy for sleep; Figure 1). It also served as a platform for setting and monitoring personal goals.

Previous studies on patients with T2D have shown more considerable impacts on physical behavior [11] and glycated hemoglobin [28] if self-monitoring was complemented with other intervention features. In the MySteps trial, self-monitoring with mHealth was supplemented with a PA leaflet devised for patients with T2D, 3 face-to-face counseling sessions, 4 telephone counseling contacts, and a YouTube video on how to increase daily walking. The features were designed by using information from former studies about the motivators and barriers of PA in a Finnish sample of adults at high risk of or diagnosed with T2D [43,44]. The timing, content, behavior change techniques (BCTs), and delivery modes of SMC are introduced in Multimedia Appendix 1. The BCTs were drawn from the Coventry, Aberdeen & London—Refined taxonomy refined for PA and healthy eating [45] and BCT taxonomy v1 [46].

#### Provider Training

The physiotherapists participated in a 4-hour training about the study protocol, measurements, and intervention contents before the first recruitment. The participant-specific counseling cards guided the physiotherapists to the protocol and contents of each intervention arm. The cards were also used in documenting the realization and duration of the sessions and contacts. At the first face-to-face session, the physiotherapists instructed the participants of the SMC and self-monitoring only group to use the accelerometer and smartphone app and to replace the accelerometer battery if necessary.

### Evaluation of Perceived Behavior Change Needs

The information on participants’ perceived behavior change needs for increasing daily walking and other nonexercise PA was collected with a questionnaire (Multimedia Appendix 2). The questionnaire was based on the capability, opportunity, motivation—behavior (COM-B) model, which identifies 3 critical sources of behavior: capability, opportunity, and motivation [47]. The model generally suggests that to achieve a change in the target behavior, the change must first happen in capability, opportunity, and/or motivation related to the target behavior or the behaviors supporting the target behavior [33]. Capability, opportunity, and motivation are further linked to the theoretical domains framework (TDF) with more detailed determinants of behavior [48]. Capability is mapped to knowledge, cognitive and interpersonal skills, memory, attention and decision processes, physical skills, and behavioral regulation. Opportunity is linked to social influences, environmental context, and resources. Motivation is driven by professional or social role and identity, beliefs about capabilities, consequences, optimism, intentions, goals, reinforcement, and emotion [33].

COM-B model with the determinants is further connected to 9 intervention functions (education, persuasion, incentivization, coercion, training, restriction, environmental restructuring, modeling, and enablement) and 7 policy categories (communication or marketing, guidelines, fiscal measures, regulation, legislation, environmental or social planning, and service provision), which represent the means for changing the target behavior [33]. The means are then delivered by BCTs, which are the smallest active ingredients of the intervention [33]. All these elements together form the Behavior Change Wheel, which is a synthesis of various behavior change models and can be used for designing and evaluating behavior change interventions [47].

The questionnaire for evaluating perceived behavior change needs in capability, opportunity, and motivation was adapted for the study purposes from the COM-B Self-Evaluation Questionnaire (COM-B-Qv1) introduced by Michie et al [33]. As recommended by the questionnaire developers, the items were specifically addressed to the target behavior, which in this study was daily walking and other nonexercise PA. The first 10 items in the questionnaire represented the need for change in capability, the next 7 items in opportunity, and the last 5 items in motivation. At the end of the questionnaire, the respondents had an option to write an item in an open space. In each item, the respondents were asked to assess with a Likert scale from 1=not at all to 5=very much how much change would be needed for them to increase daily walking and other nonexercise PA.

**Figure 2.**
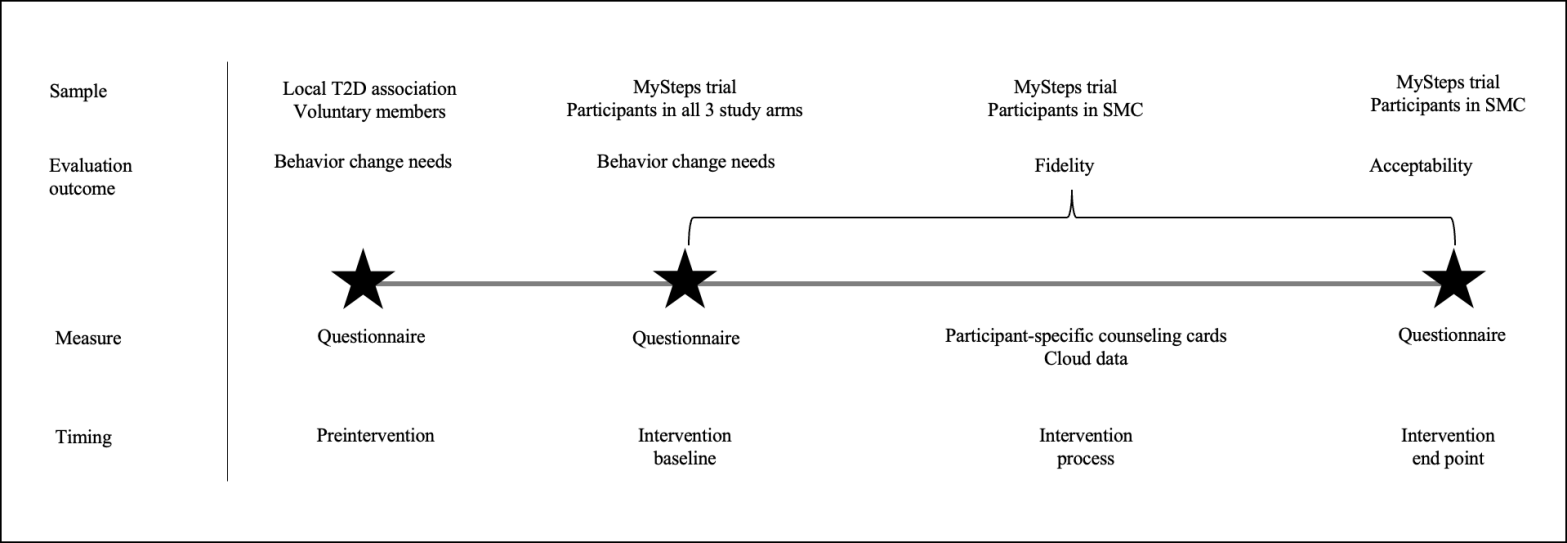
Data collection for the evaluation of the perceived behavior change needs of the patients with T2D for increasing daily walking and other nonexercise physical activity and for the evaluation of SMC’s implementation (fidelity and acceptability). SMC: self-monitoring with counseling; T2D: type 2 diabetes.

The questionnaire also included background questions on age, gender, height, weight, perceived health, and intensity-specific PA and was first targeted to the members of the local T2D association in Pirkanmaa, Finland (Figure 2). In its newsletter, the association informed its members about the possibility of participating. The members could either complete an electronic questionnaire through a link in the newsletter or go to the association’s office to complete a paper questionnaire. To increase the sample size, the same questionnaire on perceived behavior change needs was addressed to the participants of the MySteps trial as part of their baseline measurements (Figure 2).

### Implementation Evaluation

#### Overview

Proctor et al [34] have introduced 3 interrelated types of outcomes of implementation research: implementation outcomes, service outcomes, and client outcomes. This paper focuses on implementation outcomes, which, according to Proctor et al [34], include acceptability, adoption, appropriateness, costs, feasibility, fidelity, penetration, and sustainability. The same outcomes have been determined for evaluating the implementation of behavioral intervention technology [49]. However, the terminology and definitions related to the implementation outcomes are inconsistent, and the operationalization and measurement of the same outcome vary across the studies [34]. Especially, the constructs of acceptability, appropriateness, and feasibility have been used interchangeably [34].

The constructs selected for acceptability in the planning phase of this study (usefulness, ease of use, credibility, and satisfaction) considerably overlapped with the constructs of adoption, appropriateness, and feasibility introduced by Proctor et al [34] and Hermes et al [49]. Moreover, the rest of the introduced outcomes (costs, penetration, and sustainability) are considered more suitable for organization-level evaluation [34,49] and interventions aiming at larger-scale clinical uptake and maintenance. As a result, the fidelity and acceptability remained the most applicable implementation outcomes of the intervention.

#### Fidelity

Fidelity is defined as the extent to which the intervention was delivered as intended by the program developers [49,50]. The recommendations on fidelity evaluation in health behavior studies cover study design, provider training, treatment delivery, treatment receipt, and enactment of treatment skills [51]. In studies involving behavioral intervention technologies, fidelity is traditionally measured at the provider level [49]. Examples of typical fidelity measurements are protocol adherence, dose or amount of program delivered, and quality of program delivery [34].

Fidelity can be measured with observations, checklists, or self-reports [34]. A systematic review by Lewis et al [52] did not find any measures of fidelity in behavioral health interventions. A recent systematic review by Mettert et al [53] detected a rapid increase in measures across the implementation outcomes and identified 18 fidelity measures. However, the information on the psychometric properties of the instruments was poorly reported, which prevented us from making conclusions about their suitability.

In the MySteps trial, the fidelity of SMC was evaluated during the 6-month intervention (Figure 2) with a measure designed for the study purposes. The physiotherapists kept records of completed face-to-face sessions, telephone contacts, and their contents with participant-specific counseling cards. Compliance with using the accelerometer and smartphone app was obtained from the cloud data. A more specific description of fidelity evaluation is presented in Table 1.

**Table 1.** Fidelity evaluation by delivery feature in the main intervention arm (self-monitoring with counseling [SMC]).

Delivery feature	Data source
Accelerometer and smartphone app	Cloud computing
Number (%) of weekly use days (maximum of 273 days in the whole group)^a^	
Number (%) of high-engagement users (5-7 days per week)	
Number (%) of moderate-engagement users (3-4 days per week)	
Number (%) of low-engagement users (1-2 days per week)	
Number (%) of nonusers (0 days per week)	
Number (%) of participants failing to pair the accelerometer with the smartphone app (nonsync)	
Impact of the counseling sessions and telephone contacts on participants’ weekly use of the accelerometer and smartphone app	
Face-to-face counseling	Counseling cards^b^
Proportion of realized counseling sessions (maximum of 117^c^)	
Time used for all counseling sessions	
Time used for a single counseling session	
Telephone counseling	Counseling cards^b^
Proportion of realized telephone contacts (maximum of 156^c^)	
Time used for all telephone contacts	
Time used for a single telephone contact	

a Number of participants in SMC was 39, and each participant had the possibility to use the accelerometer and app on 7 days a week.

b Participant-specific card, where the nurses kept record of completed counseling sessions and contacts.

c The number of participants in SMC was 39, and each participant had to have 3 face-to-face counseling sessions and 4 telephone counseling contacts during the 6-month intervention.

#### Acceptability

Low acceptability may lead to poor fidelity and weak effectiveness [50]. Acceptability has thus been recognized as a key factor for the effectiveness of interventions [54]. Consequently, acceptability has been included in some national guidelines on conducting process evaluation in complex interventions [55]. However, the definition and operationalization of acceptability have been incoherent in guidelines and interventions, which has hampered its assessment and led to a need for developing a more theoretically grounded framework [54]. Consequently, acceptability has been defined as “a multi-faceted construct, represented by seven component constructs: effective attitude, burden, perceived effectiveness, ethicality, intervention coherence, opportunity costs, and self-efficacy” [54].

Acceptability is the most evaluated implementation outcome in health care [52,56]. A systematic review [52] discovered 50 acceptability instruments, and another systematic review [53] found 32 measures used in behavioral health care settings. However, both reviews concluded that the psychometric strength of the instruments is poorly reported, and most instruments show low psychometric quality. Moreover, those with some psychometric evidence were lengthy, which raised concerns about their clinical feasibility [52].

In the MySteps trial, the acceptability of SMC was assessed retrospectively from the participants’ viewpoint as part of 6-month end-point measurements (Figure 2). The purpose was to examine the user experiences on the contents and delivery of SMC. At the time of the study design, no ready-made and valid instrument was found for the present purpose. Thus, previous literature was used adaptively in determining the constructs of acceptability [57,58]. The constructs selected were usefulness, ease of use, credibility, and satisfaction. In each construct, the participants were asked to assess the primary features of SMC, that is, accelerometer (not applicable in credibility), feedback views of the smartphone app, face-to-face counseling sessions, and telephone counseling contacts. The response alternatives were presented on a Likert scale from 1=not at all to 5=extremely. After each construct, the participants could tell in an open space what would have improved their experience.

### Ethical Considerations

The study was conducted in accordance with the Declaration of Helsinki, the guidelines of the Finnish National Board on Research Integrity on the ethical principles of research with human participants and on the responsible conduct of research, and was approved by the research ethics committee of the Northern Savo Hospital District (running number 303/2017). The participants gave their written consents to participate after being fully informed about the ethical principles and data protection (EU General Data Protection Regulation and Finnish Data Protection Act) of the study, which cover also the means to secure the privacy and confidentiality of the participants’ data. The participants were not compensated for participating in the study. The study has been registered in the ClinicalTrials.gov database (NCT04587414).

### Statistics

Descriptive data analysis was performed, and absolute numbers, percentages, means, and SDs were provided as appropriate. Generalized linear mixed model (GLMM) with logistic regression was used to assess the effect of face-to-face counseling sessions and telephone contacts in the weeks 4 (telephone), 8 (session), 12 (telephone), and 16 (telephone) on using the app at least 3 days per week (moderate engagement). The contact in week 21 (telephone) was excluded because it was suspected that its effect may have been confounded by accelerometer battery loss. An equally long 3-week period from the preceding contact was used as a repeated measure with autoregressive-moving-average 11 covariance structure. For each participant, 4 cycles of app use were thus identified (weeks 4‐7, 8‐11, 12‐15, and 16‐19), and these cycles were applied as a random effect with a variance component covariance type. Results were reported as odds ratios and 95% CIs. Statistical analysis was done using SPSS (version 29; IBM Corp).

## Results

### Perceived Behavior Change Needs

Altogether, 144 patients with T2D completed the adapted COM-B-Qv1 questionnaire. In total, 25 of them were members of the local T2D association, and 119 were participants of the MySteps trial. The background and health-related characteristics of the respondents to the questionnaire are described in Table 2. The majority (86/141, 61%) of the respondents were women, aged 55 years or older (120/141, 85%), and overweight or obese (133/144, 92%). On average, their self-reported weekly engagement in MVPA was 139 minutes, which is slightly less than the minimum of 150 minutes recommendation for patients with T2D or healthy adults in general.

**Table 2.** Background and health-related characteristics of the respondents providing information on perceived behavioral change needs for increasing daily walking and other nonexercise physical activity.

	Preintervention^a^ (n=25)	Intervention baseline^b^ (n=119)	Total (N=144)
Age (years), mean (SD)^c^	66.2 (7.7)	60.7 (6.9)	61.7 (7.4)
Age group (years), n (%)^c^			
40-54	1 (4)	20 (17)	21 (15)
55-65	11 (44)	65 (56)	76 (54)
>65	13 (52)	31 (27)	44 (31)
Women, n (%)^c^	18 (72)	68 (59)	86 (61)
BMI (kg/m^2^), mean (SD)	30.2 (5.1)	31.8 (4.5)	31.5 (4.7)
<25	4 (16)	7 (6)	11 (8)
25-30	8 (32)	33 (28)	41 (28)
>30	13 (52)	79 (66)	92 (64)
Married or spouse, n (%)^c^	—^d^	90 (78)	—
Working status, n (%)			
Working	—	62 (53)	—
Retired	—	54 (47)	—
Perceived health, n (%)			
Fairly good or good	7 (28)	—	—
Average	15 (60)	—	—
Fairly poor or poor	3 (12)	—	—
Perceived health compared with age-mates, n (%)^c^			
Better	—	12 (10)	—
Equal	—	59 (51)	—
Worse	—	45 (39)	—
Weekly minutes of total physical activity, mean (SD)^e^	346 (211)	380 (521)	374 (480)
Light physical activity	228 (201)	237 (386)	236 (359)
Moderate-intensity physical activity	91 (85)	121 (214)	116 (198)
Vigorous-intensity physical activity	26 (46)	22 (67)	23 (63)

a Data obtained prior to the MySteps trial from the members of the local type 2 diabetes association.

b Data obtained at baseline measurements from 119/120 participants of the MySteps trial.

c Information missing from 3 respondents.

d Not available.

e Self-reported: questionnaire to the members of the local type 2 diabetes association and to the participants of the MySteps trial.

If the response alternatives 4 and 5 were combined, 3 items in capability and 2 items in motivation seemed to stand out in respondents’ behavior change needs for increasing daily walking and other nonexercise PA (Table 3). The items in capability were “Have stronger resilience against cravings, which reduce my physical activity” (91/143, 64%; mean 3.8, SD 1.2), “More easily overcome physical barriers, which reduce my physical activity” (83/140, 59%; mean 3.6, SD 1.1), and “Having more physical stamina” (75/142, 53%; mean 3.6, SD 1.2). For motivation, the items standing out similarly were “Plan my physical activity better” (80/141, 56%; mean 3.5, SD 1.1) and “Develop a routine of being physically active without having to think about it specifically” (96/142, 68%; mean 3.9, SD 1.1). However, there was only a little variation in the percentages and means of the motivational items.

**Table 3.** Behavior change needs perceived by patients with T2D^a^ displayed with numbers of respondents, distributions (%), and means^b^.

Behavior change needs	Likert scale from 1 to 5^c^					Mean score (SD)
	1, n (%)	2, n (%)	3, n (%)	4, n (%)	5, n (%)	
Capability						
Know more about the benefits of walking and other physical activity.	34 (24)	46 (32)	46 (32)	12 (8)	4 (3)	2.3 (1.0)
Know more about how to increase walking and other physical activity.	27 (19)	36 (25)	52 (37)	19 (13)	8 (6)	2.6 (1.1)
Be physically more skillful.	19 (13)	36 (25)	42 (30)	33 (23)	12 (8)	2.9 (1.2)
Learn how to reason walking and physical activity more effectively.	22 (16)	25 (18)	31 (22)	36 (26)	27 (19)	3.1 (1.3)
Be physically stronger (eg, better muscular fitness).	11 (8)	25 (18)	36 (25)	35 (25)	35 (25)	3.4 (1.2)
Have stronger resilience against cravings, which reduce my physical activity (eg, watching television, commuting by car, and preferring elevator over stairs).	7 (5)	14 (10)	31 (22)	44 (31)	47 (33)	3.8 (1.2)
More easily overcome physical barriers, which reduce my physical activity (eg, tiredness, overweight, and poor fitness).	5 (4)	19 (13)	34 (24)	47 (33)	36 (26)	3.6 (1.1)
More easily overcome mental barriers (eg, negative attitudes, images, or emotions).	15 (11)	39 (28)	41 (29)	34 (24)	12 (9)	2.9 (1.1)
Have more physical stamina (eg, better cardiovascular fitness).	8 (6)	16 (11)	43 (30)	37 (26)	38 (27)	3.6 (1.2)
Have more mental stamina (eg, better tolerance for haste and time pressure).	8 (6)	49 (35)	39 (28)	35 (25)	10 (7)	2.9 (1.1)
Opportunity						
Have more time for physical activity.	33 (23)	44 (31)	31 (22)	23 (16)	10 (7)	2.5 (1.2)
Have more money for physical activity.	63 (44)	45 (32)	24 (17)	9 (6)	1 (1)	1.9 (1.0)
Have better physical activity outfits or equipment.	51 (36)	52 (36)	26 (18)	9 (6)	5 (3)	2.1 (1.1)
Have easier access to places, which enable physical activity.	51 (36)	40 (28)	31 (22)	13 (9)	7 (5)	2.2 (1.2)
Have more people around me, who are also physically active.	26 (19)	31 (22)	46 (33)	26 (19)	11 (8)	2.8 (1.2)
Have more reminders when I need to be physically active.	13 (9)	27 (19)	44 (31)	40 (29)	16 (11)	3.1 (1.1)
Have more support from family or other important people.	30 (21)	44 (31)	37 (26)	22 (15)	10 (7)	2.6 (1.2)
Motivation						
Feel more urge to be physically active.	10 (7)	21 (15)	43 (30)	33 (23)	35 (25)	3.4 (1.2)
Worry more about not being physically active.	12 (8)	27 (19)	33 (23)	39 (27)	31 (22)	3.4 (1.3)
Sense more strongly that physical activity is good for me.	12 (8)	32 (22)	29 (20)	37 (26)	33 (23)	3.3 (1.3)
Plan my daily physical activity better.	7 (5)	19 (13)	35 (25)	54 (38)	26 (18)	3.5 (1.1)
Develop a routine of being physically active without having to think about it specifically.	4 (3)	12 (8)	30 (21)	47 (33)	49 (35)	3.9 (1.1)
Other						
Do something else, what?	16 (17)	11 (12)	34 (36)	11 (12)	23 (24)	3.1 (1.4)

a T2D: type 2 diabetes.

b The responses from the local T2D association (n=25) and from the baseline measurements of the MySteps trial (n=119) are combined (n=144). The number of responses varies from 140 to 143 in the items of capability, opportunity, and motivation and was 95 in the category “other.”

c 1 indicates that not at all change would be needed and 5 indicates that very much change would be needed to increase daily walking and other nonexercise physical activity.

### Implementation

#### Fidelity

Altogether, 39 participants of the MySteps trial were randomized into SMC after the baseline measurements. As each participant was to have 3 face-to-face counseling sessions and 4 telephone counseling contacts, the maximum number of face-to-face sessions and telephone contacts in SMC was 117 and 156, respectively.

Based on the participant-specific counseling cards, the number of realized face-to-face counseling sessions was 112 of 117 (96%). The second session was missing from 2 participants, and the third session was missing from 3 participants. The total time used for the 3 face-to-face sessions varied from 80 to 230 minutes, being on average 152 (SD 34) minutes. The mean time spent for 1 session was 51 (SD 11) minutes, ranging from 27 to 77 minutes.

The total number of realized telephone counseling contacts was 145 of 156 (93%). In total, 2 participants missed the first contact, and 3 participants missed the second, third, and fourth contacts. The total time used for the telephone counseling sessions varied from 72 to 175 minutes, being on average 109 (SD 30) minutes. The mean time spent for 1 contact was 27 (SD 8) minutes, ranging from 18 to 44 minutes.

**Figure 3.**
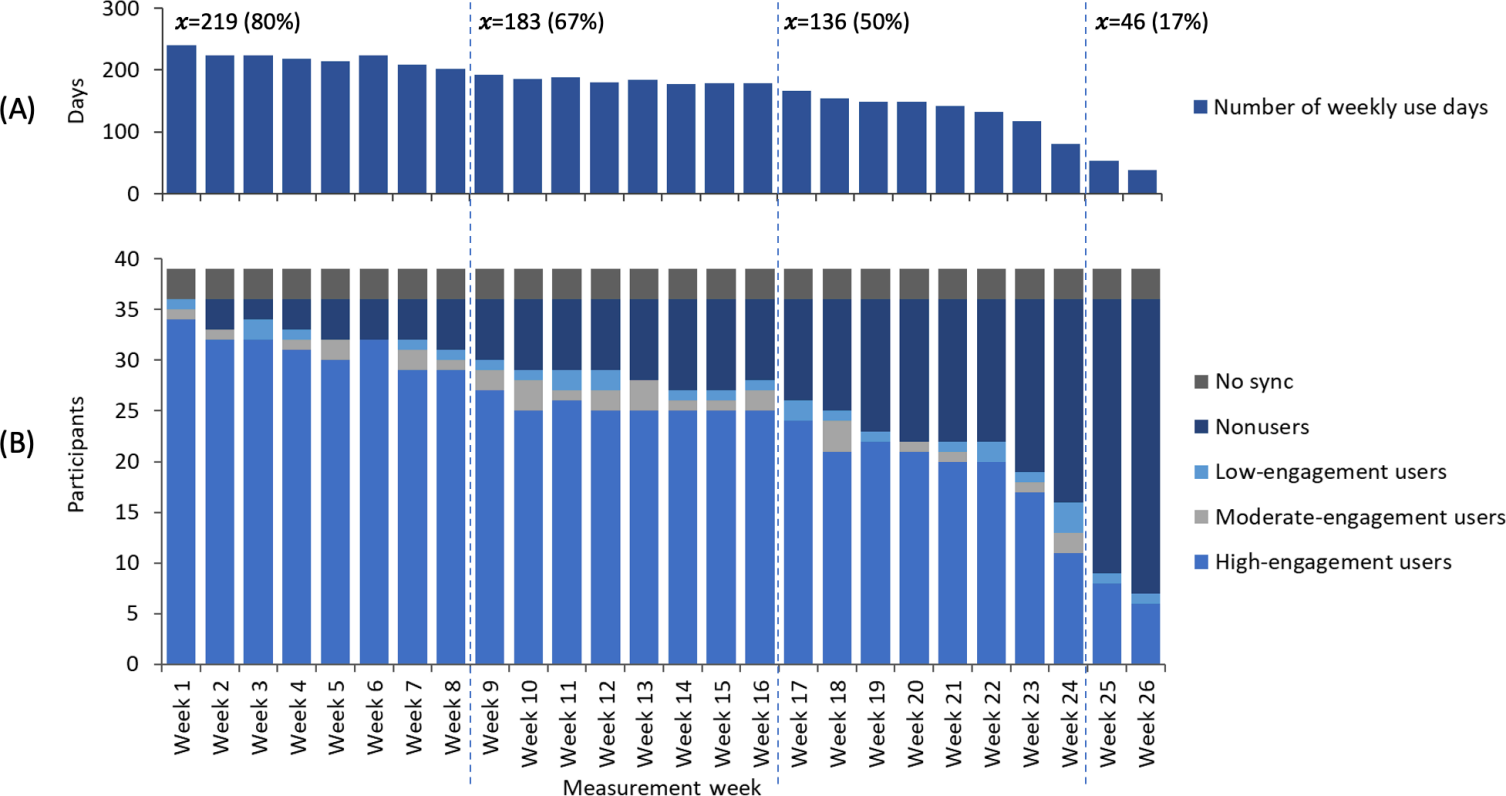
Compliance of the patients with T2D with using the accelerometer and smartphone app during the intervention weeks broken into 4 periods with dashed lines (weeks 1‐8, 9‐16, 17‐24, and 25‐26). The upper histogram (**A**) shows the mean number and percentage of weekly use days, and the lower (**B**) presents the number of participants who failed to pair the devices (no sync), used the devices on 0 days per week (nonusers), 1‐2 days per week (low engagement), 3‐4 days per week (moderate engagement), and 5‐7 days per week (high engagement).

At the beginning of the intervention, 3 (8%) participants failed to pair their accelerometer with the smartphone app (Figure 3). In the rest of the participants, the use of the devices declined moderately and steadily until intervention week 21 and dropped notably thereafter. The number of weekly use days was on average 219 (80% from the maximum of 273) during weeks 1‐8, 183 (67%) during weeks 9‐16, 136 (50%) during weeks 17‐24, and 46 (17%) during weeks 25‐26. As a result, the mean weekly accelerometer use was 54%.

In those succeeding in pairing the devices (n=36), the mean number of users was 33 (92%) during weeks 1‐8, 28 (78%) during weeks 9‐16, 22 (61%) during weeks 17‐24, and 8 (22%) during weeks 25‐26. Correspondingly, the mean number of high-engagement users was 31 (86%), 25 (69%), 20 (56%), and 7 (19%), and the mean number of nonusers was 3 (8%), 8 (22%), 14 (39%), and 28 (78%), respectively. As shown in Figure 3, the weekly number of moderate- and low-engagement participants remained quite stable throughout the intervention.

According to the GLMM analysis, the face-to-face counseling sessions and telephone contacts in weeks 4, 8, 12, and 16 seemed to affect the participants’ use of the smartphone app at least 3 days per week (moderate engagement) since the odds for lower engagement increased as the time elapsed from the contact. After the contacts, the odds ratios for using the smartphone app less than 3 days per week in the 3 following weeks increased and were 1.36 (95% CI 0.896-2.063; *P*=.15), 1.79 (95% CI 1.124-2.859; *P*=.01), and 2.49 (95% CI 1.511-4.111; *P<*.001).

#### Acceptability

A total of 34 participants in SMC (87% of the 39 participants randomized into SMC) provided information on the acceptability of SMC at the end of the MySteps trial at 6-month measurements. The background characteristics of the respondents in SMC at the end of the study did not differ from the baseline background characteristics of all participants: the majority were women (27/34, 79%), aged 55 years or older (28/34, 82%), and overweight or obese (30/34, 88%). Most participants were working (24/34, 71%) and perceived their health better or equal to their age-mates (27/34, 79%). However, at the end of the intervention, when the questionnaire on acceptability was addressed, the participants of SMC had more self-reported MVPA than all participants at baseline (mean 240 vs 139 minutes).

The construct-specific distributions (%) and means of the acceptability responses are shown in Table 4. Overall, it seems that the features of SMC were mostly acceptable to the participants. The lowest mean scores for acceptability were seen in the credibility of the feedback views and in the satisfaction related to the accelerometer.

**Table 4.** Acceptability of the features of SMC^a^ within each evaluation construct: usefulness, ease of use, credibility, and satisfaction.

Delivery modes within each evaluation construct	Distribution, n (%)					Mean score (SD)
	1=not at all	2	3	4	5=extremely	
Usefulness						
Accelerometer	2 (3)	6 (9)	6 (8)	19 (27)	37 (53)	4.2 (1.1)
Feedback views from the smartphone app	2 (3)	1 (1)	6 (9)	17 (25)	43 (62)	4.4 (0.9)
Face-to-face sessions	1 (3)	0 (0)	2 (5)	10 (28)	23 (64)	4.5 (0.8)
Telephone contacts	1 (3)	1 (3)	1 (3)	14 (39)	19 (53)	4.4 (0.9)
Ease of use						
Accelerometer	2 (3)	2 (3)	4 (6)	18 (26)	43 (62)	4.4 (0.9)
Feedback views from the smartphone app	1 (1)	2 (3)	4 (6)	11 (16)	50 (74)	4.6 (0.9)
Face-to-face sessions	0 (0)	0 (0)	2 (6)	9 (25)	25 (69)	4.6 (0.6)
Telephone contacts	0 (0)	0 (0)	0 (0)	7 (19)	29 (81)	4.8 (0.4)
Credibility						
Feedback views from the accelerometer and smartphone app	4 (6)	8 (11)	6 (9)	29 (42)	22 (32)	3.8 (1.2)
Face-to-face sessions	0 (0)	0 (0)	1 (3)	6 (17)	29 (80)	4.8 (0.5)
Telephone contacts	0 (0)	0 (0)	1 (3)	7 (19)	28 (78)	4.8 (0.5)
Satisfaction						
Accelerometer	5 (7)	4 (6)	11 (16)	23 (33)	27 (38)	3.9 (1.2)
Feedback views from the smartphone app	2 (3)	3 (4)	11 (16)	18 (26)	36 (51)	4.2 (1.0)
Face-to-face sessions	0 (0)	0 (0)	1 (3)	7 (20)	27 (77)	4.7 (0.5)
Telephone contacts	0 (0)	0 (0)	1 (3)	7 (19)	28 (78)	4.8 (0.5)

a SMC: self-monitoring with counseling.

## Discussion

### Principal Findings

First, this paper reports perceived behavior change needs for increasing daily walking and other nonexercise PA in a sample of patients with T2D. Second, the paper examines the implementation of the main intervention arm of the MySteps trial, which was designed to promote walking and other nonexercise PA among patients with T2D through an mHealth approach within a real-life health care setting. The findings may be used in developing future mHealth interventions for these patients.

The participants’ responses pointed out 3 items in capability and 2 in motivation, which stood out as perceived behavior change needs. Moreover, the main intervention arm of the MySteps trial showed good fidelity and acceptability among patients with T2D, although some challenges were also experienced.

### Perceived Behavior Change Needs

Based on the responses to the COM-B-Qv1 questionnaire, it seems that to promote walking and nonexercise PA in patients with T2D, attention should be paid especially to capability and motivation as sources of behavior. The findings are in line with previous reviews, indicating that BCTs related to opportunity such as social influences and environmental issues are not among the most effective BCTs in promoting PA or improving glucose metabolism among patients with T2D [20,28,59]. Earlier studies in healthy adults similarly imply that capability and motivation are the key drivers of PA [60]. However, in a younger population [61] and special culture groups [62], opportunity and thereby social support may play a more important role.

The perceived needs for change in capability, which scored the highest, may refer to the importance of improving the patients’ skills to resist cravings, overcome barriers, and develop the capacity to maintain physical effort. Correspondingly, the perceived needs distinguished in motivation seem to indicate the importance of strengthening the patients’ beliefs about their capabilities to make action plans and of improving habitual engagement in PA.

The present discoveries on sources of behavior and their more specific items were much more focused and limited than in a small Chinese study involving 28 individuals with metabolic syndrome [63]. Between-study differences are likely to originate from cultural and methodological disparities, meaning that contextual factors may play an important role and should be taken into consideration when interpreting the findings on behavioral analysis and comparing them between studies.

The small sample size of this study and its selectivity limit the generalizability of the findings. Thus, the findings merely reflect the views of patients with T2D, who are mostly women, aged 55 years or older, overweight, and not physically very active. However, as women with T2D take on average fewer steps than men and the daily steps tend to decrease with age [17], there seems to be a particular need for interventions targeting this group of people. In this sense, our sample represents the population most likely to benefit from PA interventions. Still, more studies involving larger and heterogenous samples are needed to build up nationally and internationally generalizable interventions for patients with T2D.

Another obvious limitation of this study concerns the COM-B model’s ability to explain PA behavior in the T2D population. Earlier studies involving healthy adults support the COM-B model’s predictive validity for MVPA [60]. In young adults, the COM-B model explained 31% of the variance in PA behavior and was able to identify barriers and enablers of PA [61]. However, to our knowledge, studies on the psychometrics of applying COM-B among patients with T2D and other types of PA have not yet been published.

The third limitation is the nonvalidated COM-B measure adopted for the study. The questionnaire was not piloted but was found functional in the first set of participants, who were the members of the local T2D association. However, the information on the construct validity of the questionnaire is lacking, making it unclear whether the questionnaire correctly evaluated the intended sources of behavior. A questionnaire based on TDF has been proven reasonably reliable and valid in measuring the determinants of general PA in university students [64]. However, no previous studies were discovered that used the COM-B alone or in combination with TDF to evaluate the perceived behavioral needs of patients with T2D for increasing PA or especially walking.

### Implementation

#### Fidelity

The counseling procedure of the main arm of the MySteps trial was implemented as intended, regardless of the COVID-19–induced challenges to patients with T2D. One reason may be that the procedure mostly included telephone contacts. Only a few sessions or contacts were missed, which suggests that the number of sessions and contacts was feasible for the participants. The mean lengths of the individual sessions and contacts also seemed reasonable in clinical practice, although there was a large variation. The large variation probably illustrates the demand for adjusting the length of the sessions and contacts according to individual needs, which may not always be executable in real-life health care settings. The findings on fidelity and length of the sessions are in line with the most comparable study to the present one, where 83% of the patients (63% with T2D and 37% with chronic obstructive pulmonary disease) received the intended intervention of 4 sessions and where most of the sessions exceeded the intended 20 minutes [65].

In the use of accelerometer and smartphone app, the number of weekly use days and number of weekly users declined during the intervention from 80% to 17% and from 92% to 22%, respectively. Similar descending trends have been found in other mHealth interventions among patients with diabetes, which show approximately 40% user rates at the end of the study [66,67]. One exception is the study by Verwey et al [65], where 88% of the patients wore the accelerometer and smartphone app until the end of the 25-week intervention period. The progressively lowering use has been recognized also in interventions involving adults without chronic diseases, although their attrition rates have been more moderate [21]. In any case, the engagement plays an important role in mHealth interventions since it has been shown that it associates with intervention effectiveness [68].

In this study, one explanation for the deep decline in use in weeks 23‐26 may be that the accelerometer battery faded, and the participants, despite the instructions, did not replace the battery since the intervention was already coming to its end. If the weeks 23 through 26 were eliminated, the average user rate for the last weeks would be 64%. In any case, the attrition rate in mHealth studies seems generally quite high and is worth considering in future studies [68].

Interestingly, while the proportion of participants with a high engagement declined from 86% to 19%, the proportion of participants with moderate and low engagement remained practically the same, and the proportion of nonusers increased from 8% to 78%. This indicates that at least in the present intervention, the participants rather stopped using the devices than just reduced their use. If this discovery applies more widely, its impact on successful implementation needs to be considered in future mHealth interventions.

The relevance of individual contacts in facilitating the use of smartphone apps is seldom examined. In this study, the findings of the GLMM analysis emphasized their importance. The discovery is somewhat contradictory to a recent systematic review, which indicates that, in general, feedback on self-monitoring might not prevent attrition from self-monitoring interventions [69]. The more comparable studies, nonetheless, show that consultations are much appreciated among patients with T2D and not substitutable with an app [39].

The fidelity evaluation would have gained from more extensive cloud-based information on the use of the accelerometer and smartphone app. Unfortunately, this information could not have been extracted from the cloud data. Earlier studies have identified similar drawbacks and highlighted the importance of close collaboration with the technology team to develop tools for collecting fidelity information from raw digital data [70].

Overall, previous research shows that the delivery of PA interventions in patients with T2D is seldom evaluated at a practical level, and usually, the process data are limitedly and inconsistently reported [36]. This is regardless of the 2004 recommendation by the National Institute of Health Behavior Change Consortium to facilitate fidelity evaluation, especially in health behavior change studies [51].

With the increasing number of web-based and mHealth interventions aiming at health behavior change, the need for improving and standardizing evaluation reports has also been identified [71]. As a result, the CONSORT-EHEALTH (Consolidated Standards of Reporting Trials of Electronic and Mobile Health Applications and Online Telehealth) guideline on reporting eHealth and mHealth interventions has been published [71]. The guideline extends various checklist items of the CONSORT (Consolidated Standards of Reporting Trials) statement for randomized controlled studies and highly recommends, for example, reporting metrics of use (checklist item 17a-i) and technological problems (checklist item 19) as intervention outcomes (Checklist 1) [71]. Nevertheless, the systematic review by Blackman et al [72] shows that external validity is poorly reported in mHealth interventions promoting PA and provides specific recommendations for more comprehensive reporting including engagement.

#### Acceptability

The findings on the acceptability of SMC are encouraging, given that the majority of participants were women, aged 55 years or older, overweight, and physically not so active. On the other hand, the present results compare to previous studies, which show that adults with T2D appreciate self-monitoring and health professional facilitation as features of digital health intervention [39,40] and that age is not necessarily a barrier to using smartphone apps [66].

However, there were indications that some improvements would be needed to strengthen the credibility of the smartphone views and satisfaction related to the accelerometer. In the open spaces of the questionnaire, the respondents brought up similar concerns indicated in the few previous studies, including the inability of the accelerometer to account for all PA such as cycling and gymnasium activities [73] and technical problems related to a wireless connection [65,74].

The findings are not comparable with previous studies because acceptability has not been evaluated in research using mobile-based technology to promote health behavior change in people with T2D [38]. In many studies, the goal of the intervention has been to improve glycemic control through more comprehensive self-management apps [29,39]. These studies show variable satisfaction ratings and multiple usability problems such as multistep tasks, limited functionality and interaction, and difficult system navigation [29]. Usability problems, in turn, have been suggested to cause the experience of failure and additional burden to the participants, which can subsequently lead to so-called digital distress in some users [39].

A larger sample size would have brought more variance to the responses and may have shown more clearly the developmental demands related to the features of SMC. Moreover, the inclusion of the health providers’ views would have deepened the understanding of acceptability, as it has been found that the technical problems, for example, are easy for the participants to manifest during counseling [73]. Providers’ perspectives may have also brought out valuable information, especially about the applicability of the digital features of the intervention to everyday practice [37]. Previous studies have identified multiple influences on the use of digital tools in primary care [75]. In addition, using a measure with strong psychometric qualities would have improved the validity of acceptability findings. However, until now, only a few such measures have been identified in health care [53,56]. The unvalidated measures and specific features of the intervention may limit the generalization of the findings on acceptability.

### Conclusions

In total, 3 items in capability and 2 in motivation stood out as perceived behavior change needs among patients with T2D for increasing walking and other nonexercise PA. The finding calls for additional research since no comparable studies have been done, to our knowledge. In addition, the explanatory value of the COM-B model and psychometric properties of the COM-B questionnaire concerning nonexercise PA in patients with T2D deserves further attention.

The main intervention arm of the MySteps mHealth trial based on self-monitoring and counseling showed good fidelity and acceptability in a sample of patients with T2D, which represented the people benefiting most from increasing PA. However, some challenges in acceptability were discovered regarding the smartphone feedback views and accelerometer use, but overall, acceptability proved better than expected, considering the special features of the sample. The challenges were quite similar to the ones observed in a few previous studies, underscoring the importance of pretesting technology-based approaches in interventions promoting PA in the T2D population.

To summarize, the findings indicate that to design scalable mHealth interventions in the future to promote nonexercise PA in patients with T2D requires (1) valid measures on perceived behavior change needs, (2) studies with larger and more heterogenous samples, and (3) more comprehensive evaluation of implementation outcomes from the perspectives of both patients and professionals.

## Supplementary material

10.2196/80304 Multimedia Appendix 1 Timing, content, behavior change techniques, and delivery modes of the main intervention arm (self-monitoring with counseling).

10.2196/80304 Multimedia Appendix 2 The questionnaire used in examining the perceived behavior change needs of the patients with type 2 diabetes in increasing walking and other nonexercise physical activity.

10.2196/80304 Checklist 1 CONSORT-EHEALTH checklist (V1.6.1).

## Abbreviations

BCT: behavior change technique
COM-B: capability, opportunity, motivation—behavior
COM-B-Qv1: COM-B Self-Evaluation Questionnaire
CONSORT: Consolidated Standards of Reporting Trials
CONSORT-EHEALTH: Consolidated Standards of Reporting Trials of Electronic and Mobile Health Applications and Online Telehealth
GLMM: generalized linear mixed model
LIPA: light-intensity physical activity
mHealth: mobile health
MVPA: moderate-to-vigorous intensity physical activity
PA: physical activity
SMC: self-monitoring with counseling
T2D: type 2 diabetes
TDF: theoretical domains framework
