# Supplementary material for: mHealth Intervention to Promote Nonexercise Physical Activity in Patients With Type 2 Diabetes: Secondary Analysis and Implementation Study
Source: JMIR Form Res. 2026 Mar 19;10:e80304. doi: 10.2196/80304 (PMC13002163; doi:10.2196/80304)
Supplement: Multimedia Appendix 1 [file formative-v10-e80304-s001.docx]

| **Week** | **Intervention content** | **Behaviour Change Techniques (BCTs)** | **Delivery mode** |
| --- | --- | --- | --- |
| 1 | **Physical activity leaflet and feedback from 6-minute walk test performed at baseline assessment:** assessing current behaviour, need for change and reasons for making a change  **Questionnaire on behaviour change needs at baseline assessment:** identifying what needs to change to increase daily walking and other free-living physical activity  **ExSed App:** giving instructions for self-monitoring and goal setting  **Discussion:** making an action plan for achieving the goals | **P**rovide information on the consequences of increasing physical activity and reducing sedentary behavior to the individual, **P**rovide instruction on how to perform the behaviour, **P**rompt self-monitoring of behaviour (items important for increasing physical activity, steps), **G**oal setting (steps), **A**ction planning (steps) | Face-to-face 1 |
| 4 | **ExSed App:** reviewing and adjusting the goals  **Discussion:** Providing feedback and updating the action plan  **YouTube-videos:** referring to demonstrations on how to increase walking | **P**rovide feedback on performance (steps), **P**rompt review of behavioural goals, **A**ction planning, **D**emonstrate the behaviour, **P**rovide instruction on how to perform the behaviour | Telephone 1 |
| 8 | **ExSed dApp:** reviewing and adjusting the goals  **Discussion:** providing feedback, updating the action plan, getting back to videos, identifying barriers overcoming barriers, identifying need for social support | **P**rovide feedback on performance (steps), **P**rompt review of behavioural goals, **A**ction planning, **P**rovide instruction on how to perform the behaviour, **B**arrier identification / problem solving, **P**lan social support | Face-to-face 2 |
| 12 | **ExSed App:** reviewing and adjusting the goals  **Discussion:** providing feedback, updating the action plan, getting back to social support, transferring success to different situations | **P**rovide feedback on performance (steps), **P**rompt review of behavioural goals, **A**ction planning, **P**rovide instruction on how to perform the behaviour, **R**elapse prevention / coping planning, **T**each to use prompts / cues, **P**rompt practice (resilience against sedentary behaviour) | Telephone 2 |
| 16 | **ExSed App:** reviewing and adjusting the goals  **Discussion:** providing feedback, updating the action plan, getting back to transferring success, identifying means to resist cravings | **P**rovide feedback on performance (steps), **P**rompt review of behavioural goals, **A**ction planning, **P**rovide instruction on how to perform the behaviour, **R**elapse prevention / coping planning, **P**rompt practice (resilience against sedentary behaviour) | Telephone 3 |
| 21 | **ExSed App:** reviewing and adjusting the goals  **Discussion:** providing feedback, updating the action plan, getting back to resisting cravings, identifying potential reasons for relapses, identifying means for preventing relapses | **P**rovide feedback on performance (steps), **P**rompt review of behavioural goals, **A**ction planning, **R**elapse prevention / coping planning, **P**rompt practice (resilience against sedentary behaviour) | Telephone 4 |
| 26 | **Physical activity leaflet:** comparing current behaviour with baseline, assessing how well the reasons for increasing physical activity were realized  **Questionnaire on behaviour change needs:** comparing the responses with baseline  **ExSed App:** reviewing and adjusting the goals  **Discussion:** providing feedback, making a long-term action plan, getting back to preventing relapses | **P**rovide feedback on performance (steps), **P**rompt review of behavioural goals, **A**ction planning, **P**rovide feedback on consequences of behaviour, **P**rovide feedback on performance (change in items important for increasing physical activity) | Face-to-face 3 |
