# Supplementary figures and images for: mHealth Intervention to Promote Nonexercise Physical Activity in Patients With Type 2 Diabetes: Secondary Analysis and Implementation Study
Source: JMIR Form Res. 2026 Mar 19;10:e80304. doi: 10.2196/80304 (PMC13002163; doi:10.2196/80304)

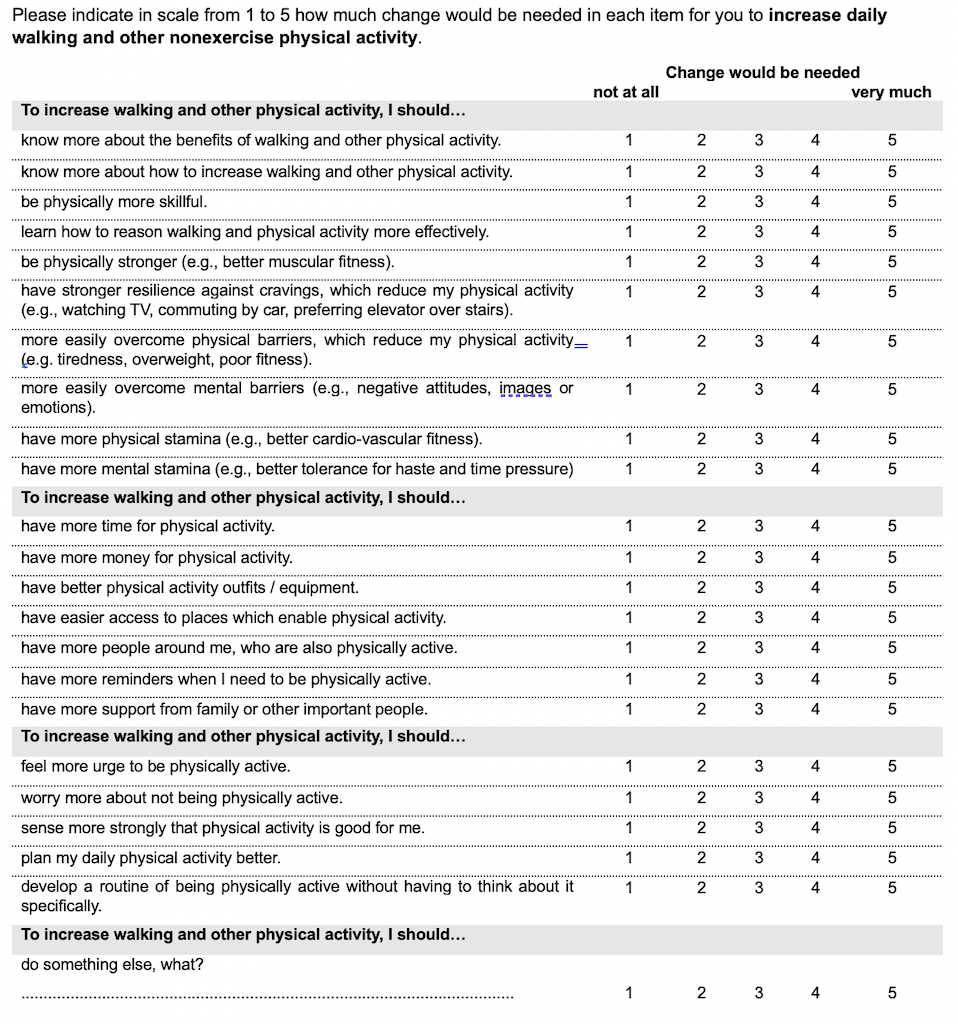

Supplement: Multimedia Appendix 2 [file formative-v10-e80304-s002.png]
