# Supplementary material for: mHealth Intervention to Promote Nonexercise Physical Activity in Patients With Type 2 Diabetes: Secondary Analysis and Implementation Study
Source: JMIR Form Res. 2026 Mar 19;10:e80304. doi: 10.2196/80304 (PMC13002163; doi:10.2196/80304)
Supplement: Checklist 1 [file formative-v10-e80304-s003.pdf]

CONSORT-EHEALTH (V 1.6.1) - Submission/Publication Form

The CONSORT-EHEALTH checklist is intended for authors of randomized trials evaluating web-based and Internet-based applications/interventions, including mobile interventions, electronic games (incl multiplayer games), social media, certain telehealth applications, and other interactive and/or networked electronic applications. Some of the items (e.g. all subitems under item 5 - description of the intervention) may also be applicable for other study designs.

The goal of the CONSORT EHEALTH checklist and guideline is to be
a) a guide for reporting for authors of RCTs,
b) to form a basis for appraisal of an ehealth trial (in terms of validity)

CONSORT-EHEALTH items/subitems are MANDATORY reporting items for studies published in the Journal of Medical Internet Research and other journals / scientific societies endorsing the checklist.

Items numbered 1., 2., 3., 4a., 4b etc are original CONSORT or CONSORT-NPT (non-pharmacologic treatment) items.
Items with Roman numerals (i., ii, iii, iv etc.) are CONSORT-EHEALTH extensions/clarifications.

As the CONSORT-EHEALTH checklist is still considered in a formative stage, we would ask that you also RATE ON A SCALE OF 1-5 how important/useful you feel each item is FOR THE PURPOSE OF THE CHECKLIST and reporting guideline (optional).

Mandatory reporting items are marked with a red *.
In the textboxes, either copy & paste the relevant sections from your manuscript into this form - please include any quotes from your manuscript in QUOTATION MARKS,
or answer directly by providing additional information not in the manuscript, or elaborating on why the item was not relevant for this study.

YOUR ANSWERS WILL BE PUBLISHED AS A SUPPLEMENTARY FILE TO YOUR PUBLICATION IN JMIR AND ARE CONSIDERED PART OF YOUR PUBLICATION (IF ACCEPTED).
Please fill in these questions diligently. Information will not be copyedited, so please use proper spelling and grammar, use correct capitalization, and avoid abbreviations.

DO NOT FORGET TO SAVE AS PDF _AND_ CLICK THE SUBMIT BUTTON SO YOUR ANSWERS ARE IN OUR DATABASE !!!

Citation Suggestion (if you append the pdf as Appendix we suggest to cite this paper in the caption):
Eysenbach G, CONSORT-EHEALTH Group
CONSORT-EHEALTH: Improving and Standardizing Evaluation Reports of Web-based and Mobile Health Interventions
J Med Internet Res 2011;13(4):e126
URL: http://www.jmir.org/2011/4/e126/
doi: 10.2196/jmir.1923
PMID: 22209829

Kirjaudu Googleen, jotta voit tallentaa edistymisesi. Lue lisää

* Pakollinen kysymys

Your name *
First Last

Minna Aittasalo

Primary Affiliation (short), City, Country *
University of Toronto, Toronto, Canada

Laurea University of Applied Sciences, Finland

Your e-mail address *
abc@gmail.com

minna.aittasalo@laurea.fi

Title of your manuscript *
Provide the (draft) title of your manuscript.

Promoting non-exercise physical activity in patients with type 2 diabetes: perceived behavior change needs and implementation of mHealth intervention

Name of your App/Software/Intervention *
If there is a short and a long/alternate name, write the short name first and add the long name in brackets.

ExSed

Evaluated Version (if any)
e.g. "V1", "Release 2017-03-01", "Version 2.0.27913"

Oma vastauksesi

Language(s) *
What language is the intervention/app in? If multiple languages are available, separate by comma (e.g. "English, French")

Finnish

URL of your Intervention Website or App
e.g. a direct link to the mobile app on app in appstore (itunes, Google Play), or URL of the website. If the intervention is a DVD or hardware, you can also link to an Amazon page.

http://www.exsed.com

URL of an image/screenshot (optional)

Oma vastauksesi

Accessibility *
Can an enduser access the intervention presently?

- ( ) access is free and open
- (•) access only for special usergroups, not open
- ( ) access is open to everyone, but requires payment/subscription/in-app purchases
- ( ) app/intervention no longer accessible
- ( ) Muu:

Primary Medical Indication/Disease/Condition *
e.g. "Stress", "Diabetes", or define the target group in brackets after the condition, e.g. "Autism (Parents of children with)", "Alzheimers (Informal Caregivers of)"

Patients with Type 2 Diabetes

Primary Outcomes measured in trial *
comma-separated list of primary outcomes reported in the trial

Perceived behavior change needs, fidelity, acc

Secondary/other outcomes
Are there any other outcomes the intervention is expected to affect?

Oma vastauksesi

Recommended "Dose" *
What do the instructions for users say on how often the app should be used?

- (•) Approximately Daily
- ( ) Approximately Weekly
- ( ) Approximately Monthly
- ( ) Approximately Yearly
- ( ) "as needed"
- ( ) Muu:

Approx. Percentage of Users (starters) still using the app as recommended after 3 months *

- ( ) unknown / not evaluated
- ( ) 0-10%
- ( ) 11-20%
- ( ) 21-30%
- ( ) 31-40%
- ( ) 41-50%
- (•) 51-60%
- ( ) 61-70%
- ( ) 71%-80%
- ( ) 81-90%
- ( ) 91-100%
- ( ) Muu:

Overall, was the app/intervention effective? *

- ( ) yes: all primary outcomes were significantly better in intervention group vs control
- ( ) partly: SOME primary outcomes were significantly better in intervention group vs control
- ( ) no statistically significant difference between control and intervention
- ( ) potentially harmful: control was significantly better than intervention in one or more outcomes
- ( ) inconclusive: more research is needed
- (•) Muu: Effectiveness was not examined in this study

Article Preparation Status/Stage *
At which stage in your article preparation are you currently (at the time you fill in this form)

- ( ) not submitted yet - in early draft status
- ( ) not submitted yet - in late draft status, just before submission
- ( ) submitted to a journal but not reviewed yet
- (•) submitted to a journal and after receiving initial reviewer comments
- ( ) submitted to a journal and accepted, but not published yet
- ( ) published
- ( ) Muu:

Journal *
If you already know where you will submit this paper (or if it is already submitted), please provide the journal name (if it is not JMIR, provide the journal name under "other")

- ( ) not submitted yet / unclear where I will submit this
- ( ) Journal of Medical Internet Research (JMIR)
- ( ) JMIR mHealth and UHealth
- ( ) JMIR Serious Games
- ( ) JMIR Mental Health
- ( ) JMIR Public Health
- (•) JMIR Formative Research
- ( ) Other JMIR sister journal
- ( ) Muu:

Is this a full powered effectiveness trial or a pilot/feasibility trial? *

- (•) Pilot/feasibility
- ( ) Fully powered

Manuscript tracking number *
If this is a JMIR submission, please provide the manuscript tracking number under "other" (The ms tracking number can be found in the submission acknowledgement email, or when you login as author in JMIR. If the paper is already published in JMIR, then the ms tracking number is the four-digit number at the end of the DOI, to be found at the bottom of each published article in JMIR)

- ( ) no ms number (yet) / not (yet) submitted to / published in JMIR
- (•) Muu: JFR ms#80304

TITLE AND ABSTRACT

1a) TITLE: Identification as a randomized trial in the title

1a) Does your paper address CONSORT item 1a? *
I.e does the title contain the phrase "Randomized Controlled Trial"? (if not, explain the reason under "other")

- ( ) yes
- (•) Muu: The trial itself is RCT but the manuscript reports secondary findings on

1a-i) Identify the mode of delivery in the title
Identify the mode of delivery. Preferably use "web-based" and/or "mobile" and/or "electronic game" in the title. Avoid ambiguous terms like "online", "virtual", "interactive". Use "Internet-based" only if Intervention includes non-web-based Internet components (e.g. email), use "computer-based" or "electronic" only if offline products are used. Use "virtual" only in the context of "virtual reality" (3-D worlds). Use "online" only in the context of "online support groups". Complement or substitute product names with broader terms for the class of products (such as "mobile" or "smart phone" instead of "iphone"), especially if the application runs on different platforms.

subitem not at all important 1 ( ) 2 ( ) 3 ( ) 4 ( ) 5 ( ) essential

Does your paper address subitem 1a-i? *
Copy and paste relevant sections from manuscript title (include quotes in quotation marks "like this" to indicate direct quotes from your manuscript), or elaborate on this item by providing additional information not in the ms, or briefly explain why the item is not applicable/relevant for your study

"implementation of mHealth intervention"

1a-ii) Non-web-based components or important co-interventions in title
Mention non-web-based components or important co-interventions in title, if any (e.g., "with telephone support").

subitem not at all important 1 ( ) 2 ( ) 3 ( ) 4 ( ) 5 ( ) essential

Does your paper address subitem 1a-ii?
Copy and paste relevant sections from manuscript title (include quotes in quotation marks "like this" to indicate direct quotes from your manuscript), or elaborate on this item by providing additional information not in the ms, or briefly explain why the item is not applicable/relevant for your study

Oma vastauksesi

1a-iii) Primary condition or target group in the title
Mention primary condition or target group in the title, if any (e.g., "for children with Type I Diabetes") Example: A Web-based and Mobile Intervention with Telephone Support for Children with Type I Diabetes: Randomized Controlled Trial

subitem not at all important 1 ( ) 2 ( ) 3 ( ) 4 ( ) 5 ( ) essential

Does your paper address subitem 1a-iii? *
Copy and paste relevant sections from manuscript title (include quotes in quotation marks "like this" to indicate direct quotes from your manuscript), or elaborate on this item by providing additional information not in the ms, or briefly explain why the item is not applicable/relevant for your study

"in patients with type 2 diabetes"

1b) ABSTRACT: Structured summary of trial design, methods, results, and conclusions
NPT extension: Description of experimental treatment, comparator, care providers, centers, and blinding status.

1b-i) Key features/functionalities/components of the intervention and comparator in the METHODS section of the ABSTRACT
Mention key features/functionalities/components of the intervention and comparator in the abstract. If possible, also mention theories and principles used for designing the site. Keep in mind the needs of systematic reviewers and indexers by including important synonyms. (Note: Only report in the abstract what the main paper is reporting. If this information is missing from the main body of text, consider adding it)

subitem not at all important 1 ( ) 2 ( ) 3 ( ) 4 ( ) 5 ( ) essential

Does your paper address subitem 1b-i? *
Copy and paste relevant sections from the manuscript abstract (include quotes in quotation marks "like this" to indicate direct quotes from your manuscript), or elaborate on this item by providing additional information not in the ms, or briefly explain why the item is not applicable/relevant for your study

"included 24-hour accelerometer use, smartphone application with personal feedback, a PA leaflet, a YouTube video on walking, and individual counseling with three face-to-face sessions and four telephone contacts"

1b-ii) Level of human involvement in the METHODS section of the ABSTRACT
Clarify the level of human involvement in the abstract, e.g., use phrases like "fully automated" vs. "therapist/nurse/care provider/physician-assisted" (mention number and expertise of providers involved, if any). (Note: Only report in the abstract what the main paper is reporting. If this information is missing from the main body of text, consider adding it)

subitem not at all important 1 ( ) 2 ( ) 3 ( ) 4 ( ) 5 ( ) essential

Does your paper address subitem 1b-ii?
Copy and paste relevant sections from the manuscript abstract (include quotes in quotation marks "like this" to indicate direct quotes from your manuscript), or elaborate on this item by providing additional information not in the ms, or briefly explain why the item is not applicable/relevant for your study

"individual counseling with three face-to-face sessions and four telephone contacts"

1b-iii) Open vs. closed, web-based (self-assessment) vs. face-to-face assessments in the METHODS section of the ABSTRACT
Mention how participants were recruited (online vs. offline), e.g., from an open access website or from a clinic or a closed online user group (closed usergroup trial), and clarify if this was a purely web-based trial, or there were face-to-face components (as part of the intervention or for assessment). Clearly say if outcomes were self-assessed through questionnaires (as common in web-based trials). Note: In traditional offline trials, an open trial (open-label trial) is a type of clinical trial in which both the researchers and participants know which treatment is being administered. To avoid confusion, use "blinded" or "unblinded" to indicated the level of blinding instead of "open", as "open" in web-based trials usually refers to "open access" (i.e. participants can self-enrol). (Note: Only report in the abstract what the main paper is reporting. If this information is missing from the main body of text, consider adding it)

subitem not at all important 1 ( ) 2 ( ) 3 ( ) 4 ( ) 5 ( ) essential

Does your paper address subitem 1b-iii?
Copy and paste relevant sections from the manuscript abstract (include quotes in quotation marks "like this" to indicate direct quotes from your manuscript), or elaborate on this item by providing additional information not in the ms, or briefly explain why the item is not applicable/relevant for your study

"Data on fidelity was accumulated during the intervention through counseling cards and cloud computing. Data on acceptability was collected with a questionnaire at the end of the intervention."

1b-iv) RESULTS section in abstract must contain use data
Report number of participants enrolled/assessed in each group, the use/uptake of the intervention (e.g., attrition/adherence metrics, use over time, number of logins etc.), in addition to primary/secondary outcomes. (Note: Only report in the abstract what the main paper is reporting. If this information is missing from the main body of text, consider adding it)

subitem not at all important 1 ( ) 2 ( ) 3 ( ) 4 ( ) 5 ( ) essential

Does your paper address subitem 1b-iv?
Copy and paste relevant sections from the manuscript abstract (include quotes in quotation marks "like this" to indicate direct quotes from your manuscript), or elaborate on this item by providing additional information not in the ms, or briefly explain why the item is not applicable/relevant for your study

"from a separate sample of patients with T2D (n=25) and at the intervention baseline (n=119)"

1b-v) CONCLUSIONS/DISCUSSION in abstract for negative trials
Conclusions/Discussions in abstract for negative trials: Discuss the primary outcome - if the trial is negative (primary outcome not changed), and the intervention was not used, discuss whether negative results are attributable to lack of uptake and discuss reasons. (Note: Only report in the abstract what the main paper is reporting. If this information is missing from the main body of text, consider adding it)

subitem not at all important 1 ( ) 2 ( ) 3 ( ) 4 ( ) 5 ( ) essential

Does your paper address subitem 1b-v?
Copy and paste relevant sections from the manuscript abstract (include quotes in quotation marks "like this" to indicate direct quotes from your manuscript), or elaborate on this item by providing additional information not in the ms, or briefly explain why the item is not applicable/relevant for your study

"However, the challenges discovered underscore the importance of pre-testing technology-based approaches in patients with T2D."

INTRODUCTION

2a) In INTRODUCTION: Scientific background and explanation of rationale

2a-i) Problem and the type of system/solution
Describe the problem and the type of system/solution that is object of the study: intended as stand-alone intervention vs. incorporated in broader health care program? Intended for a particular patient population? Goals of the intervention, e.g., being more cost-effective to other interventions, replace or complement other solutions? (Note: Details about the intervention are provided in "Methods" under 5)

subitem not at all important 1 ( ) 2 ( ) 3 ( ) 4 ( ) 5 ( ) essential

Does your paper address subitem 2a-i? *
Copy and paste relevant sections from the manuscript (include quotes in quotation marks "like this" to indicate direct quotes from your manuscript), or elaborate on this item by providing additional information not in the ms, or briefly explain why the item is not applicable/relevant for your study

"The purpose of this study is to examine i) the perceived behaviour change needs of patients with T2D for increasing daily walking and other non-exercise PA and ii) the implementation of an mHealth approach to increase patients' daily walking and other non-exercise PA. These were the secondary aims of the clinical intervention trial. The main aim was to examine the effectiveness of the trial in increasing the daily steps of the patients, which was, however, compromised due to the outbreak of COVID-19 pandemic in 2020. Thus, only the results concerning the secondary aims are reported here. The findings may be utilised in designing future mHealth interventions for patients with T2D."

2a-ii) Scientific background, rationale: What is known about the (type of) system
Scientific background, rationale: What is known about the (type of) system that is the object of the study (be sure to discuss the use of similar systems for other conditions/diagnoses, if appropiate), motivation for the study, i.e. what are the reasons for and what is the context for this specific study, from which stakeholder viewpoint is the study performed, potential impact of findings [2]. Briefly justify the choice of the comparator.

subitem not at all important 1 ( ) 2 ( ) 3 ( ) 4 ( ) 5 ( ) essential

Does your paper address subitem 2a-ii? *
Copy and paste relevant sections from the manuscript (include quotes in quotation marks "like this" to indicate direct quotes from your manuscript), or elaborate on this item by providing additional information not in the ms, or briefly explain why the item is not applicable/relevant for your study

"Despite its importance, implementation is inconsistently and insufficiently reported in interventions promoting PA in patients with T2D [36]. As part of the implementation, a notable gap exists in research concerning the perceptions of patients with T2D about mHealth technology [37-39]. Previous studies indicate large variability in the usability of mHealth applications among patients with T2D [29,40] and that satisfaction can improve the adoption of technology-assisted interventions [41]."

2b) In INTRODUCTION: Specific objectives or hypotheses

Does your paper address CONSORT subitem 2b? *
Copy and paste relevant sections from the manuscript (include quotes in quotation marks "like this" to indicate direct quotes from your manuscript), or elaborate on this item by providing additional information not in the ms, or briefly explain why the item is not applicable/relevant for your study

"The purpose of this study is to examine i) the perceived behaviour change needs of patients with T2D for increasing daily walking and other non-exercise PA and ii) the implementation of an mHealth approach to increase patients' daily walking and other non-exercise PA. These were the secondary aims of the clinical intervention trial. The main aim was to examine the effectiveness of the trial in increasing the daily steps of the patients, which was, however, compromised due to the outbreak of COVID-19 pandemic in 2020. Thus, only the results concerning the secondary aims are reported here."

METHODS

3a) Description of trial design (such as parallel, factorial) including allocation ratio

Does your paper address CONSORT subitem 3a? *
Copy and paste relevant sections from the manuscript (include quotes in quotation marks "like this" to indicate direct quotes from your manuscript), or elaborate on this item by providing additional information not in the ms, or briefly explain why the item is not applicable/relevant for your study

"After the baseline measurements the participants were randomized into three arms: i) self-monitoring with counseling (SMC), ii) self-monitoring only, and iii) usual care. SMC was considered the primary intervention arm of the study and is the focus of the implementation evaluation of this paper."

3b) Important changes to methods after trial commencement (such as eligibility criteria), with reasons

Does your paper address CONSORT subitem 3b? *
Copy and paste relevant sections from the manuscript (include quotes in quotation marks "like this" to indicate direct quotes from your manuscript), or elaborate on this item by providing additional information not in the ms, or briefly explain why the item is not applicable/relevant for your study

No changes were made to the methods after trial commencement.

3b-i) Bug fixes, Downtimes, Content Changes
Bug fixes, Downtimes, Content Changes: ehealth systems are often dynamic systems. A description of changes to methods therefore also includes important changes made on the intervention or comparator during the trial (e.g., major bug fixes or changes in the functionality or content) (5-iii) and other "unexpected events" that may have influenced study design such as staff changes, system failures/downtimes, etc. [2].

	1	2	3	4	5	
subitem not at all important	○	○	○	○	○	essential

Does your paper address subitem 3b-i?

Copy and paste relevant sections from the manuscript (include quotes in quotation marks "like this" to indicate direct quotes from your manuscript), or elaborate on this item by providing additional information not in the ms, or briefly explain why the item is not applicable/relevant for your study

"The fidelity evaluation would have gained from more extensive cloud-based information on the use of the accelerometer and smartphone application. Unfortunately, this information could not have been extracted from the cloud data. Earlier studies have identified similar drawbacks and highlighted the importance of close collaboration with the technology team to develop tools for collecting fidelity information from raw digital data [70]."

4a) Eligibility criteria for participants

Does your paper address CONSORT subitem 4a? *

Copy and paste relevant sections from the manuscript (include quotes in quotation marks "like this" to indicate direct quotes from your manuscript), or elaborate on this item by providing additional information not in the ms, or briefly explain why the item is not applicable/relevant for your study

"Inclusion criteria for participation were age 20—69 years, BMI <40 kg/m2, not meeting the current PA recommendation for health, and the ability to conduct a 6-minute walk test [42]. Exclusion criteria for participation were any problem that limited the ability to walk without an aid, any health problem that hindered participation in PA, and inability or unwillingness to use a smartphone or wear an accelerometer."

4a-i) Computer / Internet literacy

Computer / Internet literacy is often an implicit "de facto" eligibility criterion - this should be explicitly clarified.

	1	2	3	4	5	
subitem not at all important	○	○	○	○	○	essential

Does your paper address subitem 4a-i?

Copy and paste relevant sections from the manuscript (include quotes in quotation marks "like this" to indicate direct quotes from your manuscript), or elaborate on this item by providing additional information not in the ms, or briefly explain why the item is not applicable/relevant for your study

"Exclusion criteria for participation were any problem that limited the ability to walk without an aid, any health problem that hindered participation in PA, and inability or unwillingness to use a smartphone or wear an accelerometer."

4a-ii) Open vs. closed, web-based vs. face-to-face assessments:

Open vs. closed, web-based vs. face-to-face assessments: Mention how participants were recruited (online vs. offline), e.g., from an open access website or from a clinic, and clarify if this was a purely web-based trial, or there were face-to-face components (as part of the intervention or for assessment), i.e., to what degree got the study team to know the participant. In online-only trials, clarify if participants were quasi-anonymous and whether having multiple identities was possible or whether technical or logistical measures (e.g., cookies, email confirmation, phone calls) were used to detect/prevent these.

	1	2	3	4	5	
subitem not at all important	○	○	○	○	○	essential

Does your paper address subitem 4a-ii? *

Copy and paste relevant sections from the manuscript (include quotes in quotation marks "like this" to indicate direct quotes from your manuscript), or elaborate on this item by providing additional information not in the ms, or briefly explain why the item is not applicable/relevant for your study

"Two physiotherapists recruited the patients with T2D for the MySteps trial from the primary care units of the Siun Sote area and through a local newspaper."

4a-iii) Information giving during recruitment

Information given during recruitment. Specify how participants were briefed for recruitment and in the informed consent procedures (e.g., publish the informed consent documentation as appendix, see also item X26), as this information may have an effect on user self-selection, user expectation and may also bias results.

	1	2	3	4	5	
subitem not at all important	○	○	○	○	○	essential

Does your paper address subitem 4a-iii?

Copy and paste relevant sections from the manuscript (include quotes in quotation marks "like this" to indicate direct quotes from your manuscript), or elaborate on this item by providing additional information not in the ms, or briefly explain why the item is not applicable/relevant for your study

"The participants gave their written consents to participate after being fully informed about the ethical principles and data protection of the study."

4b) Settings and locations where the data were collected

Does your paper address CONSORT subitem 4b? *

Copy and paste relevant sections from the manuscript (include quotes in quotation marks "like this" to indicate direct quotes from your manuscript), or elaborate on this item by providing additional information not in the ms, or briefly explain why the item is not applicable/relevant for your study

"Two physiotherapists recruited the patients with T2D for the MySteps trial from the primary care units of the Siun Sote area and through a local newspaper."

4b-i) Report if outcomes were (self-)assessed through online questionnaires

Clearly report if outcomes were (self-)assessed through online questionnaires (as common in web-based trials) or otherwise.

	1	2	3	4	5	
subitem not at all important	○	○	○	○	○	essential

Does your paper address subitem 4b-i? *

Copy and paste relevant sections from the manuscript (include quotes in quotation marks "like this" to indicate direct quotes from your manuscript), or elaborate on this item by providing additional information not in the ms, or briefly explain why the item is not applicable/relevant for your study

"As part of the baseline measurements, the participants completed a questionnaire, which among other things, included questions on intensity-specific PA, and perceived behaviour change needs for increasing daily walking and other non-exercise PA. The information on participants' perceived behaviour change needs for increasing daily walking and other non-exercise PA was collected with a questionnaire (Figure 2). The questionnaire was based on the COM-B model, which identifies three critical sources of behaviour: Capability, Opportunity, and Motivation [47]."

"In the MySteps trial, the fidelity of SMC was evaluated during the 6-month intervention (Figure 3) with a measure designed for the study purposes. The physiotherapists kept records of completed face-to-face sessions, telephone contacts, and their contents with participant-specific counseling cards. Compliance with using the accelerometer and smartphone application was obtained from the cloud data. A more specific description of fidelity evaluation is presented in Table 1."

"In the MySteps trial, the acceptability of SMC was assessed retrospectively from the participants' viewpoint as part of 6-month endpoint measurements (Figure 3). The purpose was to examine the user experiences on the contents and delivery of SMC. At the time of the study design, no ready-made and valid instrument was found for the present purpose. Thus, previous literature was utilized adaptively in determining the constructs of acceptability [e.g., 57,58]. The constructs selected were usefulness, ease of use, credibility, and satisfaction. In each construct, the participants were asked to assess the primary features of SMC; ie., accelerometer (not applicable in credibility), feedback views of the smartphone application, face-to-face counseling sessions, and telephone counseling contacts. The response alternatives were presented on a Likert scale from 1 (not at all) to 5 (extremely). After each construct, the participants could tell in an open space what would have improved their experience."

4b-ii) Report how institutional affiliations are displayed

Report how institutional affiliations are displayed to potential participants [on ehealth media], as affiliations with prestigious hospitals or universities may affect volunteer rates, use, and reactions with regards to an intervention.(Not a required item – describe only if this may bias results)

	1	2	3	4	5	
subitem not at all important	○	○	○	○	○	essential

Does your paper address subitem 4b-ii?

Copy and paste relevant sections from the manuscript (include quotes in quotation marks "like this" to indicate direct quotes from your manuscript), or elaborate on this item by providing additional information not in the ms, or briefly explain why the item is not applicable/relevant for your study

The patients were recruited from the primary care units of one area so the affiliations did not play essential role in volunteer rates.

5) The interventions for each group with sufficient details to allow replication, including how and when they were actually administered

5-i) Mention names, credential, affiliations of the developers, sponsors, and owners

Mention names, credential, affiliations of the developers, sponsors, and owners [6] (if authors/evaluators are owners or developer of the software, this needs to be declared in a "Conflict of interest" section or mentioned elsewhere in the manuscript).

	1	2	3	4	5	
subitem not at all important	○	○	○	○	○	essential

Does your paper address subitem 5-i?

Copy and paste relevant sections from the manuscript (include quotes in quotation marks "like this" to indicate direct quotes from your manuscript), or elaborate on this item by providing additional information not in the ms, or briefly explain why the item is not applicable/relevant for your study

The authors/evaluators are not owners or developer of the software.

5-ii) Describe the history/development process

Describe the history/development process of the application and previous formative evaluations (e.g., focus groups, usability testing), as these will have an impact on adoption/use rates and help with interpreting results.

	1	2	3	4	5	
subitem not at all important	○	○	○	○	○	essential

Does your paper address subitem 5-ii?

Copy and paste relevant sections from the manuscript (include quotes in quotation marks "like this" to indicate direct quotes from your manuscript), or elaborate on this item by providing additional information not in the ms, or briefly explain why the item is not applicable/relevant for your study

The scientific background behind the system is shortly described on www.exsed.com.

5-iii) Revisions and updating

Revisions and updating. Clearly mention the date and/or version number of the application/intervention (and comparator, if applicable) evaluated, or describe whether the intervention underwent major changes during the evaluation process, or whether the development and/or content was "frozen" during the trial. Describe dynamic components such as news feeds or changing content which may have an impact on the replicability of the intervention (for unexpected events see item 3b).

	1	2	3	4	5	
subitem not at all important	○	○	○	○	○	essential

Does your paper address subitem 5-iii?

Copy and paste relevant sections from the manuscript (include quotes in quotation marks "like this" to indicate direct quotes from your manuscript), or elaborate on this item by providing additional information not in the ms, or briefly explain why the item is not applicable/relevant for your study

"Self-monitoring in SMC was built around a mHealth system, which included an accelerometer (MoveSense, Suunto Ltd, Finland) and a smartphone application (ExSed 2, UKK Terveyspalvelut Oy, Finland) for 26 weeks." No changes were made to the system during the study.

5-iv) Quality assurance methods

Provide information on quality assurance methods to ensure accuracy and quality of information provided [1], if applicable.

	1	2	3	4	5	
subitem not at all important	○	○	○	○	○	essential

Does your paper address subitem 5-iv?

Copy and paste relevant sections from the manuscript (include quotes in quotation marks "like this" to indicate direct quotes from your manuscript), or elaborate on this item by providing additional information not in the ms, or briefly explain why the item is not applicable/relevant for your study

The physiotherapists were in contact with the patients and ensured the quality and accuracy of information.

5-v) Ensure replicability by publishing the source code, and/or providing screenshots/screen-capture video, and/or providing flowcharts of the algorithms used

Ensure replicability by publishing the source code, and/or providing screenshots/screen-capture video, and/or providing flowcharts of the algorithms used. Replicability (i.e., other researchers should in principle be able to replicate the study) is a hallmark of scientific reporting.

	1	2	3	4	5	
subitem not at all important	○	○	○	○	○	essential

Does your paper address subitem 5-v?

Copy and paste relevant sections from the manuscript (include quotes in quotation marks "like this" to indicate direct quotes from your manuscript), or elaborate on this item by providing additional information not in the ms, or briefly explain why the item is not applicable/relevant for your study

The manuscript provides references on ExSed, which can be utilized in replication.

5-vi) Digital preservation

Digital preservation: Provide the URL of the application, but as the intervention is likely to change or disappear over the course of the years; also make sure the intervention is archived (Internet Archive, webcitation.org, and/or publishing the source code or screenshots/videos alongside the article). As pages behind login screens cannot be archived, consider creating demo pages which are accessible without login.

	1	2	3	4	5	
subitem not at all important	○	○	○	○	○	essential

Does your paper address subitem 5-vi?

Copy and paste relevant sections from the manuscript (include quotes in quotation marks "like this" to indicate direct quotes from your manuscript), or elaborate on this item by providing additional information not in the ms, or briefly explain why the item is not applicable/relevant for your study

"Self-monitoring in SMC was built around a mHealth system, which included an accelerometer (MoveSense, Suunto Ltd, Finland) and a smartphone application (ExSed 2, UKK Terveyspalvelut Oy, Finland) for 26 weeks. The scientific background behind the system is shortly described on www.exsed.com."

5-vii) Access

Access: Describe how participants accessed the application, in what setting/context, if they had to pay (or were paid) or not, whether they had to be a member of specific group. If known, describe how participants obtained "access to the platform and Internet" [1]. To ensure access for editors/reviewers/readers, consider to provide a "backdoor" login account or demo mode for reviewers/readers to explore the application (also important for archiving purposes, see vi).

	1	2	3	4	5	
subitem not at all important	○	○	○	○	○	essential

Does your paper address subitem 5-vii? *

Copy and paste relevant sections from the manuscript (include quotes in quotation marks "like this" to indicate direct quotes from your manuscript), or elaborate on this item by providing additional information not in the ms, or briefly explain why the item is not applicable/relevant for your study

"At the first face-to-face session, the physiotherapists instructed the participants of the SMC and self-monitoring only group to use the accelerometer and smartphone application and to replace the accelerometer battery if necessary."

5-viii) Mode of delivery, features/functionalities/components of the intervention and comparator, and the theoretical framework

Describe mode of delivery, features/functionalities/components of the intervention and comparator, and the theoretical framework [6] used to design them (instructional strategy [1], behaviour change techniques, persuasive features, etc., see e.g., [7, 8] for terminology). This includes an in-depth description of the content (including where it is coming from and who developed it) [1]," whether [and how] it is tailored to individual circumstances and allows users to track their progress and receive feedback" [6]. This also includes a description of communication delivery channels and – if computer-mediated communication is a component – whether communication was synchronous or asynchronous [6]. It also includes information on presentation strategies [1], including page design principles, average amount of text on pages, presence of hyperlinks to other resources, etc. [1].

	1	2	3	4	5	
subitem not at all important	○	○	○	○	○	essential

Does your paper address subitem 5-viii? *

Copy and paste relevant sections from the manuscript (include quotes in quotation marks "like this" to indicate direct quotes from your manuscript), or elaborate on this item by providing additional information not in the ms, or briefly explain why the item is not applicable/relevant for your study

"The features were designed by utilizing information from former studies about the motivators and barriers of PA in a Finnish sample of adults at high risk of or diagnosed with T2D [43-44]. The timing, content, behaviour change techniques (BCTs), and delivery modes of SMC are introduced in Appendix 1. The BCTs were drawn from the CALO-RE taxonomy refined for PA and healthy eating [45], and BCT taxonomy v1 [46]."

"The information on participants' perceived behaviour change needs for increasing daily walking and other non-exercise PA was collected with a questionnaire (Figure 2). The questionnaire was based on the COM-B model, which identifies three critical sources of behaviour: Capability, Opportunity, and Motivation [47]. The model generally suggests that to achieve a change in the target behaviour, the change must first happen in Capability, Opportunity, and/or Motivation related to the target behaviour or the behaviours supporting the target behaviour [33]. Capability, Opportunity, and Motivation are further linked to the Theoretical Domains Framework (TDF) with more detailed determinants of behaviour [48]. Capability is mapped to knowledge, cognitive and interpersonal skills, memory, attention and decision processes, physical skills, and behavioural regulation. Opportunity is linked to social influences, environmental context, and resources. Motivation is driven by professional /social role and identity, beliefs about capabilities, consequences, optimism, intentions, goals, reinforcement, and emotion [33]."

"This paper focuses on implementation outcomes, which according to Proctor et al. [34], include acceptability, adoption, appropriateness, costs, feasibility, fidelity, penetration, and sustainability."

5-ix) Describe use parameters

Describe use parameters (e.g., intended "doses" and optimal timing for use). Clarify what instructions or recommendations were given to the user, e.g., regarding timing, frequency, heaviness of use, if any, or was the intervention used ad libitum.

	1	2	3	4	5	
subitem not at all important	○	○	○	○	○	essential

Does your paper address subitem 5-ix?

Copy and paste relevant sections from the manuscript (include quotes in quotation marks "like this" to indicate direct quotes from your manuscript), or elaborate on this item by providing additional information not in the ms, or briefly explain why the item is not applicable/relevant for your study

"In the MySteps trial, the fidelity of SMC was evaluated during the 6-month intervention (Figure 3) with a measure designed for the study purposes. The physiotherapists kept records of completed face-to-face sessions, telephone contacts, and their contents with participant-specific counseling cards. Compliance with using the accelerometer and smartphone application was obtained from the cloud data."

5-x) Clarify the level of human involvement

Clarify the level of human involvement (care providers or health professionals, also technical assistance) in the e-intervention or as co-intervention (detail number and expertise of professionals involved, if any, as well as "type of assistance offered, the timing and frequency of the support, how it is initiated, and the medium by which the assistance is delivered". It may be necessary to distinguish between the level of human involvement required for the trial, and the level of human involvement required for a routine application outside of a RCT setting (discuss under item 21 – generalizability).

	1	2	3	4	5	
subitem not at all important	○	○	○	○	○	essential

Does your paper address subitem 5-x?

Copy and paste relevant sections from the manuscript (include quotes in quotation marks "like this" to indicate direct quotes from your manuscript), or elaborate on this item by providing additional information not in the ms, or briefly explain why the item is not applicable/relevant for your study

"In the MySteps trial, self-monitoring with mHealth was supplemented with a PA leaflet devised for patients with T2D, three face-to-face counseling sessions, four telephone counseling contacts, and a YouTube video on how to increase daily walking. The features were designed by utilizing information from former studies about the motivators and barriers of PA in a Finnish sample of adults at high risk of or diagnosed with T2D [43-44]. The timing, content, behaviour change techniques (BCTs), and delivery modes of SMC are introduced in Appendix 1."

5-xi) Report any prompts/reminders used

Report any prompts/reminders used: Clarify if there were prompts (letters, emails, phone calls, SMS) to use the application, what triggered them, frequency etc. It may be necessary to distinguish between the level of prompts/reminders required for the trial, and the level of prompts/reminders for a routine application outside of a RCT setting (discuss under item 21 – generalizability).

	1	2	3	4	5	
subitem not at all important	○	○	○	○	○	essential

Does your paper address subitem 5-xi? *

Copy and paste relevant sections from the manuscript (include quotes in quotation marks "like this" to indicate direct quotes from your manuscript), or elaborate on this item by providing additional information not in the ms, or briefly explain why the item is not applicable/relevant for your study

"In the MySteps trial, self-monitoring with mHealth was supplemented with a PA leaflet devised for patients with T2D, three face-to-face counseling sessions, four telephone counseling contacts, and a YouTube video on how to increase daily walking."

5-xii) Describe any co-interventions (incl. training/support)

Describe any co-interventions (incl. training/support): Clearly state any interventions that are provided in addition to the targeted eHealth intervention, as ehealth intervention may not be designed as stand-alone intervention. This includes training sessions and support [1]. It may be necessary to distinguish between the level of training required for the trial, and the level of training for a routine application outside of a RCT setting (discuss under item 21 – generalizability.

	1	2	3	4	5	
subitem not at all important	○	○	○	○	○	essential

Does your paper address subitem 5-xii? *

Copy and paste relevant sections from the manuscript (include quotes in quotation marks "like this" to indicate direct quotes from your manuscript), or elaborate on this item by providing additional information not in the ms, or briefly explain why the item is not applicable/relevant for your study

"The physiotherapists participated in a 4-hour training about the study protocol, measurements, and intervention contents before the first recruitment."

6a) Completely defined pre-specified primary and secondary outcome measures, including how and when they were assessed

Does your paper address CONSORT subitem 6a? *

Copy and paste relevant sections from the manuscript (include quotes in quotation marks "like this" to indicate direct quotes from your manuscript), or elaborate on this item by providing additional information not in the ms, or briefly explain why the item is not applicable/relevant for your study

"The information on participants' perceived behaviour change needs for increasing daily walking and other non-exercise PA was collected with a questionnaire (Figure 2). The questionnaire was based on the COM-B model, which identifies three critical sources of behaviour: Capability, Opportunity, and Motivation [47]."

"In the MySteps trial, the fidelity of SMC was evaluated during the 6-month intervention (Figure 3) with a measure designed for the study purposes. The physiotherapists kept records of completed face-to-face sessions, telephone contacts, and their contents with participant-specific counseling cards. Compliance with using the accelerometer and smartphone application was obtained from the cloud data."

"In the MySteps trial, the acceptability of SMC was assessed retrospectively from the participants' viewpoint as part of 6-month endpoint measurements (Figure 3). The purpose was to examine the user experiences on the contents and delivery of SMC. At the time of the study design, no ready-made and valid instrument was found for the present purpose. Thus, previous literature was utilized adaptively in determining the constructs of acceptability [e.g., 57,58]. The constructs selected were usefulness, ease of use, credibility, and satisfaction. In each construct, the participants were asked to assess the primary features of SMC; ie., accelerometer (not applicable in credibility), feedback views of the smartphone application, face-to-face counseling sessions, and telephone counseling contacts. The response alternatives were presented on a Likert scale from 1 (not at all) to 5 (extremely). After each construct, the participants could tell in an open space what would have improved their experience."

6a-i) Online questionnaires: describe if they were validated for online use and apply CHERRIES items to describe how the questionnaires were designed/deployed

If outcomes were obtained through online questionnaires, describe if they were validated for online use and apply CHERRIES items to describe how the questionnaires were designed/deployed [9].

	1	2	3	4	5	
subitem not at all important	○	○	○	○	○	essential

Does your paper address subitem 6a-i?

Copy and paste relevant sections from manuscript text

The outcomes were not obtained through online questionnaires.

6a-ii) Describe whether and how "use" (including intensity of use/dosage) was defined/measured/monitored

Describe whether and how "use" (including intensity of use/dosage) was defined/measured/monitored (logins, logfile analysis, etc.). Use/adoption metrics are important process outcomes that should be reported in any ehealth trial.

	1	2	3	4	5	
subitem not at all important	○	○	○	○	○	essential

Does your paper address subitem 6a-ii?

Copy and paste relevant sections from manuscript text

"In the MySteps trial, the fidelity of SMC was evaluated during the 6-month intervention (Figure 3) with a measure designed for the study purposes. The physiotherapists kept records of completed face-to-face sessions, telephone contacts, and their contents with participant-specific counseling cards. Compliance with using the accelerometer and smartphone application was obtained from the cloud data."

6a-iii) Describe whether, how, and when qualitative feedback from participants was obtained

Describe whether, how, and when qualitative feedback from participants was obtained (e.g., through emails, feedback forms, interviews, focus groups).

	1	2	3	4	5	
subitem not at all important	○	○	○	○	○	essential

Does your paper address subitem 6a-iii?

Copy and paste relevant sections from manuscript text

"In the MySteps trial, the acceptability of SMC was assessed retrospectively from the participants' viewpoint as part of 6-month endpoint measurements (Figure 3). The purpose was to examine the user experiences on the contents and delivery of SMC. At the time of the study design, no ready-made and valid instrument was found for the present purpose. Thus, previous literature was utilized adaptively in determining the constructs of acceptability [e.g., 57,58]. The constructs selected were usefulness, ease of use, credibility, and satisfaction. In each construct, the participants were asked to assess the primary features of SMC; ie., accelerometer (not applicable in credibility), feedback views of the smartphone application, face-to-face counseling sessions, and telephone counseling contacts. The response alternatives were presented on a Likert scale from 1 (not at all) to 5 (extremely). After each construct, the participants could tell in an open space what would have improved their experience."

6b) Any changes to trial outcomes after the trial commenced, with reasons

Does your paper address CONSORT subitem 6b? *

Copy and paste relevant sections from the manuscript (include quotes in quotation marks "like this" to indicate direct quotes from your manuscript), or elaborate on this item by

providing additional information not in the ms, or briefly explain why the item is not applicable/relevant for your study

No changes were made to the outcomes after the trial commenced.

7a) How sample size was determined
NPT: When applicable, details of whether and how the clustering by care provides or centers was addressed

7a-i) Describe whether and how expected attrition was taken into account when calculating the sample size
Describe whether and how expected attrition was taken into account when calculating the sample size.

	1	2	3	4	5	
subitem not at all important	○	○	○	○	○	essential

Does your paper address subitem 7a-i?
Copy and paste relevant sections from manuscript title (include quotes in quotation marks "like this" to indicate direct quotes from your manuscript), or elaborate on this item by providing additional information not in the ms, or briefly explain why the item is not applicable/relevant for your study

Oma vastauksesi

7b) When applicable, explanation of any interim analyses and stopping guidelines

Does your paper address CONSORT subitem 7b? *
Copy and paste relevant sections from the manuscript (include quotes in quotation marks "like this" to indicate direct quotes from your manuscript), or elaborate on this item by providing additional information not in the ms, or briefly explain why the item is not applicable/relevant for your study

Interim analyses and stopping guidelines were not applicable.

8a) Method used to generate the random allocation sequence
NPT: When applicable, how care providers were allocated to each trial group

Does your paper address CONSORT subitem 8a? *
Copy and paste relevant sections from the manuscript (include quotes in quotation marks "like this" to indicate direct quotes from your manuscript), or elaborate on this item by providing additional information not in the ms, or briefly explain why the item is not applicable/relevant for your study

The providers were the same for all trial groups.

8b) Type of randomisation; details of any restriction (such as blocking and block size)

Does your paper address CONSORT subitem 8b? *
Copy and paste relevant sections from the manuscript (include quotes in quotation marks "like this" to indicate direct quotes from your manuscript), or elaborate on this item by providing additional information not in the ms, or briefly explain why the item is not applicable/relevant for your study

The manuscript does not examine the effectiveness of the trial so no randomization procedure or results are reported.

9) Mechanism used to implement the random allocation sequence (such as sequentially numbered containers), describing any steps taken to conceal the sequence until interventions were assigned

Does your paper address CONSORT subitem 9? *
Copy and paste relevant sections from the manuscript (include quotes in quotation marks "like this" to indicate direct quotes from your manuscript), or elaborate on this item by providing additional information not in the ms, or briefly explain why the item is not applicable/relevant for your study

The manuscript does not examine the effectiveness of the trial so no randomization procedure or results are reported.

10) Who generated the random allocation sequence, who enrolled participants, and who assigned participants to interventions

Does your paper address CONSORT subitem 10? *
Copy and paste relevant sections from the manuscript (include quotes in quotation marks "like this" to indicate direct quotes from your manuscript), or elaborate on this item by providing additional information not in the ms, or briefly explain why the item is not applicable/relevant for your study

The manuscript does not examine the effectiveness of the trial so no randomization procedure or results are reported.

11a) If done, who was blinded after assignment to interventions (for example, participants, care providers, those assessing outcomes) and how
NPT: Whether or not administering co-interventions were blinded to group assignment

11a-i) Specify who was blinded, and who wasn't
Specify who was blinded, and who wasn't. Usually, in web-based trials it is not possible to blind the participants [1, 3] (this should be clearly acknowledged), but it may be possible to blind outcome assessors, those doing data analysis or those administering co-interventions (if any).

	1	2	3	4	5	
subitem not at all important	○	○	○	○	○	essential

Does your paper address subitem 11a-i? *
Copy and paste relevant sections from the manuscript (include quotes in quotation marks "like this" to indicate direct quotes from your manuscript), or elaborate on this item by providing additional information not in the ms, or briefly explain why the item is not applicable/relevant for your study

The manuscript does not examine the effectiveness of the trial so blinding is not relevant in the manuscript.

11a-ii) Discuss e.g., whether participants knew which intervention was the "intervention of interest" and which one was the "comparator"
Informed consent procedures (4a-ii) can create biases and certain expectations - discuss e.g., whether participants knew which intervention was the "intervention of interest" and which one was the "comparator".

	1	2	3	4	5	
subitem not at all important	○	○	○	○	○	essential

Does your paper address subitem 11a-ii?
Copy and paste relevant sections from the manuscript (include quotes in quotation marks "like this" to indicate direct quotes from your manuscript), or elaborate on this item by providing additional information not in the ms, or briefly explain why the item is not applicable/relevant for your study

The manuscript does not examine the effectiveness of the trial so participants' knowledge about the group is not relevant in the manuscript.

11b) If relevant, description of the similarity of interventions
(this item is usually not relevant for ehealth trials as it refers to similarity of a placebo or sham intervention to a active medication/intervention)

Does your paper address CONSORT subitem 11b? *
Copy and paste relevant sections from the manuscript (include quotes in quotation marks "like this" to indicate direct quotes from your manuscript), or elaborate on this item by providing additional information not in the ms, or briefly explain why the item is not applicable/relevant for your study

The manuscript does not examine the effectiveness of the trial so the similarity of the interventions is not relevant in the manuscript.

12a) Statistical methods used to compare groups for primary and secondary outcomes
NPT: When applicable, details of whether and how the clustering by care providers or centers was addressed

Does your paper address CONSORT subitem 12a? *
Copy and paste relevant sections from the manuscript (include quotes in quotation marks "like this" to indicate direct quotes from your manuscript), or elaborate on this item by providing additional information not in the ms, or briefly explain why the item is not applicable/relevant for your study

The manuscript does not examine the effectiveness of the trial so the statistical methods for group comparison are not relevant in the manuscript.

12a-i) Imputation techniques to deal with attrition / missing values
Imputation techniques to deal with attrition / missing values: Not all participants will use the intervention/comparator as intended and attrition is typically high in ehealth trials. Specify how participants who did not use the application or dropped out from the trial were treated in the statistical analysis (a complete case analysis is strongly discouraged, and simple imputation techniques such as LOCF may also be problematic [4]).

	1	2	3	4	5	
subitem not at all important	○	○	○	○	○	essential

Does your paper address subitem 12a-i? *
Copy and paste relevant sections from the manuscript (include quotes in quotation marks "like this" to indicate direct quotes from your manuscript), or elaborate on this item by providing additional information not in the ms, or briefly explain why the item is not applicable/relevant for your study

The manuscript does not examine the effectiveness of the trial so imputation techniques were not relevant in the manuscript.

12b) Methods for additional analyses, such as subgroup analyses and adjusted analyses

Does your paper address CONSORT subitem 12b? *
Copy and paste relevant sections from the manuscript (include quotes in quotation marks "like this" to indicate direct quotes from your manuscript), or elaborate on this item by providing additional information not in the ms, or briefly explain why the item is not applicable/relevant for your study

The manuscript does not examine the effectiveness of the trial so subgroup or adjusted analysis are not relevant in the manuscript.

X26) REB/IRB Approval and Ethical Considerations [recommended as subheading under "Methods"] (not a CONSORT item)

X26-i) Comment on ethics committee approval

	1	2	3	4	5	
subitem not at all important	○	○	○	○	○	essential

Does your paper address subitem X26-i?
Copy and paste relevant sections from the manuscript (include quotes in quotation marks "like this" to indicate direct quotes from your manuscript), or elaborate on this item by providing additional information not in the ms, or briefly explain why the item is not applicable/relevant for your study

"The study was conducted in accordance with the Declaration of Helsinki and was approved by The Research Ethics Committee of the Northern Savo Hospital District (running number 303/2017). The participants gave their written consents to participate after being fully informed about the ethical principles and data protection of the study. The study has been registered in ClinicalTrials database (NCT04587414)."

x26-ii) Outline informed consent procedures
Outline informed consent procedures e.g., if consent was obtained offline or online (how? Checkbox, etc.?), and what information was provided (see 4a-ii). See [6] for some items to be included in informed consent documents.

	1	2	3	4	5	
subitem not at all important	○	○	○	○	○	essential

Does your paper address subitem X26-ii?
Copy and paste relevant sections from the manuscript (include quotes in quotation marks "like this" to indicate direct quotes from your manuscript), or elaborate on this item by providing additional information not in the ms, or briefly explain why the item is not applicable/relevant for your study

The manuscript does not examine the effectiveness of the trial so outlining the consent procedure is not essential in the manuscript.

X26-iii) Safety and security procedures
Safety and security procedures, incl. privacy considerations, and any steps taken to reduce the likelihood or detection of harm (e.g., education and training, availability of a hotline)

	1	2	3	4	5	
subitem not at all important	○	○	○	○	○	essential

Does your paper address subitem X26-iii?
Copy and paste relevant sections from the manuscript (include quotes in quotation marks "like this" to indicate direct quotes from your manuscript), or elaborate on this item by providing additional information not in the ms, or briefly explain why the item is not applicable/relevant for your study

The participants were in regular contact with the physiotherapists and were able to report any harmful events to them. Also, only non-exercise physical activity comprising mostly of walking was promoted, which was unlikely to cause adverse effects to the participants.

RESULTS

13a) For each group, the numbers of participants who were randomly assigned, received intended treatment, and were analysed for the primary outcome
NPT: The number of care providers or centers performing the intervention in each group and the number of patients treated by each care provider in each center

Does your paper address CONSORT subitem 13a? *
Copy and paste relevant sections from the manuscript (include quotes in quotation marks "like this" to indicate direct quotes from your manuscript), or elaborate on this item by providing additional information not in the ms, or briefly explain why the item is not applicable/relevant for your study

"Altogether 144 patients with T2D completed the adapted COM-B-Qv1 questionnaire. Twenty-five of them were the members of the local T2D association and 119 were the participants of the MySteps trial."

"Altogether 39 participants of the MySteps trial were randomized into SMC after the baseline measurements. As each participant was to have three face-to-face counseling sessions and four telephone counseling contacts, the maximum number of face-to-face sessions and telephone contacts in SMC was 117 and 156, respectively."

"A total of 34 participants in SMC (87% of the 39 participants randomized into SMC) provided the information on the acceptability of SMC at the end of the MySteps trial at 6-month measurements."

13b) For each group, losses and exclusions after randomisation, together with reasons

Does your paper address CONSORT subitem 13b? (NOTE: Preferably, this is shown in a CONSORT flow diagram) *
Copy and paste relevant sections from the manuscript (include quotes in quotation marks "like this" to indicate direct quotes from your manuscript), or elaborate on this item by providing additional information not in the ms, or briefly explain why the item is not applicable/relevant for your study

The manuscript does not examine the effectiveness of the trial so the drop-out rates with reasoning were not relevant in the manuscript although they are reported in relation to implementation outcomes.

13b-i) Attrition diagram
Strongly recommended: An attrition diagram (e.g., proportion of participants still logging in or using the intervention/comparator in each group plotted over time, similar to a survival curve) or other figures or tables demonstrating usage/dose/engagement.

	1	2	3	4	5	
subitem not at all important	○	○	○	○	○	essential

Does your paper address subitem 13b-i?
Copy and paste relevant sections from the manuscript or cite the figure number if applicable (include quotes in quotation marks "like this" to indicate direct quotes from your manuscript), or elaborate on this item by providing additional information not in the ms, or briefly explain why the item is not applicable/relevant for your study

"At the beginning of the intervention, three (8%) participants failed to pair their accelerometer with the smartphone application (Figure 4). In the rest of the participants, the use of the devices declined moderately and steadily until intervention week 21 and dropped notably thereafter. The number of weekly usage days was on average 219 (80% from the maximum of 273) during weeks 1-8, 183 (67%) during weeks 9-16, 136 (50%) during weeks 17-24, and 46 (17%) during weeks 25-26. In those succeeding in pairing the devices (n=36), the mean number of users was 33 (92%) during weeks 1-8, 28 (78%) during weeks 9-16, 22 (61%) during weeks 17-24, and 8 (22%) during weeks 25-26. Correspondingly, the mean number of high-engagement users was 31 (86%), 25 (69%), 20 (56%) and 7 (19%) and the mean number of non-users 3 (8%), 8 (22%), 14 (39%) and 28 (78%). As shown in figure 4, the weekly number of moderate- and low-engagement participants remained quite stable throughout the intervention."

14a) Dates defining the periods of recruitment and follow-up

Does your paper address CONSORT subitem 14a? *
Copy and paste relevant sections from the manuscript (include quotes in quotation marks "like this" to indicate direct quotes from your manuscript), or elaborate on this item by providing additional information not in the ms, or briefly explain why the item is not applicable/relevant for your study

The manuscript does not examine the effectiveness of the trial so the periods of recruitment are not considered relevant. The length of the intervention itself has been indicated in the manuscript as well as the timepoints for evaluating the perceived behavior change needs, fidelity and acceptability.

14a-i) Indicate if critical "secular events" fell into the study period
Indicate if critical "secular events" fell into the study period, e.g., significant changes in Internet resources available or "changes in computer hardware or Internet delivery resources"

	1	2	3	4	5	
subitem not at all important	○	○	○	○	○	essential

Does your paper address subitem 14a-i?
Copy and paste relevant sections from the manuscript (include quotes in quotation marks "like this" to indicate direct quotes from your manuscript), or elaborate on this item by providing additional information not in the ms, or briefly explain why the item is not applicable/relevant for your study

The critical secular event of COVID-19 fell into the study period, which has been mentioned in the introduction section and which prevented from examining the effectiveness of the intervention.

14b) Why the trial ended or was stopped (early)

Does your paper address CONSORT subitem 14b? *
Copy and paste relevant sections from the manuscript (include quotes in quotation marks "like this" to indicate direct quotes from your manuscript), or elaborate on this item by providing additional information not in the ms, or briefly explain why the item is not applicable/relevant for your study

The trial lasted as long as it was intended regardless of COVID-19.

15) A table showing baseline demographic and clinical characteristics for each group
NPT: When applicable, a description of care providers (case volume, qualification, expertise, etc.) and centers (volume) in each group

Does your paper address CONSORT subitem 15? *
Copy and paste relevant sections from the manuscript (include quotes in quotation marks "like this" to indicate direct quotes from your manuscript), or elaborate on this item by providing additional information not in the ms, or briefly explain why the item is not applicable/relevant for your study

"The background and health-related characteristics of the respondents to the questionnaire are described in Table 2. Majority (61%) of the respondents were women, aged 55 years or older (85%), and overweight or obese (92%). On average, their self-reported weekly engagement in MVPA was 139 minutes, which is slightly less than the minimum recommendation for patients with T2D or healthy adults in general."

15-i) Report demographics associated with digital divide issues
In ehealth trials it is particularly important to report demographics associated with digital divide issues, such as age, education, gender, social-economic status, computer/Internet/ehealth literacy of the participants, if known.

	1	2	3	4	5	
subitem not at all important	○	○	○	○	○	essential

Does your paper address subitem 15-i? *
Copy and paste relevant sections from the manuscript (include quotes in quotation marks "like this" to indicate direct quotes from your manuscript), or elaborate on this item by providing additional information not in the ms, or briefly explain why the item is not applicable/relevant for your study

"Majority (61%) of the respondents were women, aged 55 years or older (85%), and overweight or obese (92%). On average, their self-reported weekly engagement in MVPA was 139 minutes, which is slightly less than the minimum recommendation for patients with T2D or healthy adults in general."

16) For each group, number of participants (denominator) included in each analysis and whether the analysis was by original assigned groups

16-i) Report multiple "denominators" and provide definitions
Report multiple "denominators" and provide definitions: Report N's (and effect sizes) "across a range of study participation [and use] thresholds" [1], e.g., N exposed, N consented, N used more than x times, N used more than y weeks, N participants "used" the intervention/comparator at specific pre-defined time points of interest (in absolute and relative numbers per group). Always clearly define "use" of the intervention.

	1	2	3	4	5	
subitem not at all important	○	○	○	○	○	essential

Does your paper address subitem 16-i? *
Copy and paste relevant sections from the manuscript (include quotes in quotation marks "like this" to indicate direct quotes from your manuscript), or elaborate on this item by providing additional information not in the ms, or briefly explain why the item is not applicable/relevant for your study

"Twenty-five of them were the members of the local T2D association and 119 were the participants of the MySteps trial."

"Based on the participant-specific counseling cards, the number of realized face-to-face counseling sessions was 112 out of 117 (96%).

"The second session was missing from two and the third session from three participants. The total time used for the three face-to-face sessions varied from 80 to 230 minutes being on average 152 (SD 34) minutes. The mean time spent for one session was 51 (SD 11) minutes ranging from 27 to 77 minutes. "The total number of realized telephone counseling contacts was 145 out of 156 (93%). Two participants missed the first contact and three participants the second, third and fourth contact. The total time used for the telephone counseling sessions varied from 72 to 175 minutes being on average 109 (SD 30) minutes. The mean time spent for one contact was 27 (SD 8) minutes ranging from 18 to 44 minutes."

" At the beginning of the intervention, three (8%) participants failed to pair their accelerometer with the smartphone application (Figure 4). In the rest of the participants, the use of the devices declined moderately and steadily until intervention week 21 and dropped notably thereafter. The number of weekly usage days was on average 219 (80% from the maximum of 273) during weeks 1-8, 183 (67%) during weeks 9-16, 136 (50%) during weeks 17-24, and 46 (17%) during weeks 25-26. In those succeeding in pairing the devices (n=36), the mean number of users was 33 (92%) during weeks 1-8, 28 (78%) during weeks 9-16, 22 (61%) during weeks 17-24, and 8 (22%) during weeks 25-26. Correspondingly, the mean number of high-engagement users was 31 (86%), 25 (69%), 20 (56%) and 7 (19%) and the mean number of non-users 3 (8%), 8 (22%), 14 (39%) and 28 (78%). As shown in figure 4, the weekly number of moderate- and low-engagement participants remained quite stable throughout the intervention."

16-ii) Primary analysis should be intent-to-treat
Primary analysis should be intent-to-treat, secondary analyses could include comparing only "users", with the appropriate caveats that this is no longer a randomized sample (see 18-i).

	1	2	3	4	5	
subitem not at all important	○	○	○	○	○	essential

Does your paper address subitem 16-ii?
Copy and paste relevant sections from the manuscript (include quotes in quotation marks "like this" to indicate direct quotes from your manuscript), or elaborate on this item by providing additional information not in the ms, or briefly explain why the item is not applicable/relevant for your study

The manuscript does not examine the effectiveness of the trial so the intention-to-treat principle is not relevant in the manuscript.

17a) For each primary and secondary outcome, results for each group, and the estimated effect size and its precision (such as 95% confidence interval)

Does your paper address CONSORT subitem 17a? *
Copy and paste relevant sections from the manuscript (include quotes in quotation marks "like this" to indicate direct quotes from your manuscript), or elaborate on this item by providing additional information not in the ms, or briefly explain why the item is not applicable/relevant for your study

The manuscript does not examine the effectiveness of the trial so the issues are not relevant in the manuscript.

17a-i) Presentation of process outcomes such as metrics of use and intensity of use
In addition to primary/secondary (clinical) outcomes, the presentation of process outcomes such as metrics of use and intensity of use (dose, exposure) and their operational definitions is critical. This does not only refer to metrics of attrition (13-b) (often a binary variable), but also to more continuous exposure metrics such as "average session length". These must be accompanied by a technical description how a metric like a "session" is defined (e.g., timeout after idle time) [1] (report under item 6a).

	1	2	3	4	5	
subitem not at all important	○	○	○	○	○	essential

Does your paper address subitem 17a-i?
Copy and paste relevant sections from the manuscript (include quotes in quotation marks "like this" to indicate direct quotes from your manuscript), or elaborate on this item by providing additional information not in the ms, or briefly explain why the item is not applicable/relevant for your study

Yes, these are the main outcomes reported in the manuscript and constitute most of the results section.

17b) For binary outcomes, presentation of both absolute and relative effect sizes is recommended

Does your paper address CONSORT subitem 17b? *
Copy and paste relevant sections from the manuscript (include quotes in quotation marks "like this" to indicate direct quotes from your manuscript), or elaborate on this item by providing additional information not in the ms, or briefly explain why the item is not applicable/relevant for your study

The manuscript does not examine the effectiveness of the trial so the issues are not relevant in the manuscript.

18) Results of any other analyses performed, including subgroup analyses and adjusted analyses, distinguishing pre-specified from exploratory

Does your paper address CONSORT subitem 18? *
Copy and paste relevant sections from the manuscript (include quotes in quotation marks "like this" to indicate direct quotes from your manuscript), or elaborate on this item by providing additional information not in the ms, or briefly explain why the item is not applicable/relevant for your study

The manuscript does not examine the effectiveness of the trial so the issues are not relevant in the manuscript.

18-i) Subgroup analysis of comparing only users

A subgroup analysis of comparing only users is not uncommon in ehealth trials, but if done, it must be stressed that this is a self-selected sample and no longer an unbiased sample from a randomized trial (see 16-iii).

	1	2	3	4	5	
subitem not at all important	○	○	○	○	○	essential

Does your paper address subitem 18-i?

Copy and paste relevant sections from the manuscript (include quotes in quotation marks "like this" to indicate direct quotes from your manuscript), or elaborate on this item by providing additional information not in the ms, or briefly explain why the item is not applicable/relevant for your study

The manuscript reports the findings on non-users and users. The users are furthermore classified into low-, moderate- and high-engagement groups.

19) All important harms or unintended effects in each group

(for specific guidance see CONSORT for harms)

Does your paper address CONSORT subitem 19? *

Copy and paste relevant sections from the manuscript (include quotes in quotation marks "like this" to indicate direct quotes from your manuscript), or elaborate on this item by providing additional information not in the ms, or briefly explain why the item is not applicable/relevant for your study

No harms or unintended effects due to non-exercise PA were expected.

19-i) Include privacy breaches, technical problems

Include privacy breaches, technical problems. This does not only include physical "harm" to participants, but also incidents such as perceived or real privacy breaches [1], technical problems, and other unexpected/unintended incidents. "Unintended effects" also includes unintended positive effects [2].

	1	2	3	4	5	
subitem not at all important	○	○	○	○	○	essential

Does your paper address subitem 19-i?

Copy and paste relevant sections from the manuscript (include quotes in quotation marks "like this" to indicate direct quotes from your manuscript), or elaborate on this item by providing additional information not in the ms, or briefly explain why the item is not applicable/relevant for your study

The findings on acceptability included also participants reports on technical problems. "Overall, it seems that the features of SMC were mostly acceptable to the participants. The lowest mean scores for acceptability were seen in the credibility of the feedback views and in the satisfaction related to the accelerometer."

19-ii) Include qualitative feedback from participants or observations from staff/researchers

Include qualitative feedback from participants or observations from staff/researchers, if available, on strengths and shortcomings of the application, especially if they point to unintended/unexpected effects or uses. This includes (if available) reasons for why people did or did not use the application as intended by the developers.

	1	2	3	4	5	
subitem not at all important	○	○	○	○	○	essential

Does your paper address subitem 19-ii?

Copy and paste relevant sections from the manuscript (include quotes in quotation marks "like this" to indicate direct quotes from your manuscript), or elaborate on this item by providing additional information not in the ms, or briefly explain why the item is not applicable/relevant for your study

The findings on acceptability included also participants reports on technical problems. "Overall, it seems that the features of SMC were mostly acceptable to the participants. The lowest mean scores for acceptability were seen in the credibility of the feedback views and in the satisfaction related to the accelerometer."

DISCUSSION

22) Interpretation consistent with results, balancing benefits and harms, and considering other relevant evidence

NPT: In addition, take into account the choice of the comparator, lack of or partial blinding, and unequal expertise of care providers or centers in each group

22-i) Restate study questions and summarize the answers suggested by the data, starting with primary outcomes and process outcomes (use)

Restate study questions and summarize the answers suggested by the data, starting with primary outcomes and process outcomes (use).

	1	2	3	4	5	
subitem not at all important	○	○	○	○	○	essential

Does your paper address subitem 22-i? *

Copy and paste relevant sections from the manuscript (include quotes in quotation marks "like this" to indicate direct quotes from your manuscript), or elaborate on this item by providing additional information not in the ms, or briefly explain why the item is not applicable/relevant for your study

"This paper reports perceived behavior change needs for increasing daily walking and other non-exercise PA in a sample of patients with T2D. Secondly, the paper examines the implementation of the main intervention arm of the MySteps trial, which was designed to promote walking and other non-exercise PA among patients with T2D through a mHealth approach within a real-life healthcare setting. The findings may be utilized in developing future mHealth interventions for these patients."

22-ii) Highlight unanswered new questions, suggest future research

Highlight unanswered new questions, suggest future research.

	1	2	3	4	5	
subitem not at all important	○	○	○	○	○	essential

Does your paper address subitem 22-ii?

Copy and paste relevant sections from the manuscript (include quotes in quotation marks "like this" to indicate direct quotes from your manuscript), or elaborate on this item by providing additional information not in the ms, or briefly explain why the item is not applicable/relevant for your study

"To summarize, the findings indicate that to design scalable mHealth interventions in the future to promote non-exercise PA in patients with T2D requires 1) valid measures on perceived behavior change needs, 2) studies with larger and more heterogenous samples and 3) more comprehensive evaluation of implementation outcomes from the perspectives of both patients and professionals."

20) Trial limitations, addressing sources of potential bias, imprecision, and, if relevant, multiplicity of analyses

20-i) Typical limitations in ehealth trials

Typical limitations in ehealth trials: Participants in ehealth trials are rarely blinded. Ehealth trials often look at a multiplicity of outcomes, increasing risk for a Type I error. Discuss biases due to non-use of the intervention/usability issues, biases through informed consent procedures, unexpected events.

	1	2	3	4	5	
subitem not at all important	○	○	○	○	○	essential

Does your paper address subitem 20-i? *

Copy and paste relevant sections from the manuscript (include quotes in quotation marks "like this" to indicate direct quotes from your manuscript), or elaborate on this item by providing additional information not in the ms, or briefly explain why the item is not applicable/relevant for your study

All the issues have been discussed, for example:

"The small sample size of the present study and its selectivity limit the generalizability of the findings. Thus, the findings merely reflect the views of patients with T2D, who are mostly female, aged 55 or older, overweight, and not physically very active. However, as women with T2D take on average fewer steps than men and the daily steps tend to decrease with age [17] there seems to be a particular need for interventions targeting this group of people. In this sense, the present sample represents the population most likely to benefit from PA interventions. Still, more studies involving larger and heterogenous samples are needed to build up nationally and internationally generalizable interventions for patients with T2D.

Another obvious limitation of the present study concerns the COM-B model's ability to explain PA behaviour in the T2D population. Earlier studies involving healthy adults support the COM-B model's predictive validity for MVPA [60]. In young adults, the COM-B model explained 31% of variance in PA behaviour and was able to identify barriers and enablers of PA [61]. However, to our knowledge, studies on the psychometrics of applying COM-B among patients with T2D and other types of PA have not yet been published.

The third limitation is the non-validated COM-B measure adopted for the study. The questionnaire was not piloted but was found functional in the first set of participants, who were the members of the local T2D association. However, the information on the construct validity of the questionnaire is lacking making it unclear whether the questionnaire correctly evaluated the intended sources of behaviour. A questionnaire based on TDF has been proven reasonably reliable and valid in measuring the determinants of general PA in university students [64]. However, no previous studies were discovered that used the COM-B alone or in combination with TDF to evaluate the perceived behavioural needs of patients with T2D for increasing PA or especially walking."

21) Generalisability (external validity, applicability) of the trial findings

NPT: External validity of the trial findings according to the intervention, comparators, patients, and care providers or centers involved in the trial

21-i) Generalizability to other populations

Generalizability to other populations: In particular, discuss generalizability to a general Internet population, outside of a RCT setting, and general patient population, including applicability of the study results for other organizations

	1	2	3	4	5	
subitem not at all important	○	○	○	○	○	essential

Does your paper address subitem 21-i?

Copy and paste relevant sections from the manuscript (include quotes in quotation marks "like this" to indicate direct quotes from your manuscript), or elaborate on this item by providing additional information not in the ms, or briefly explain why the item is not applicable/relevant for your study

"In this sense, the present sample represents the population most likely to benefit from PA interventions. Still, more studies involving larger and heterogenous samples are needed to build up nationally and internationally generalizable interventions for patients with T2D."

21-ii) Discuss if there were elements in the RCT that would be different in a routine application setting

Discuss if there were elements in the RCT that would be different in a routine application setting (e.g., prompts/reminders, more human involvement, training sessions or other co-interventions) and what impact the omission of these elements could have on use, adoption, or outcomes if the intervention is applied outside of a RCT setting.

	1	2	3	4	5	
subitem not at all important	○	○	○	○	○	essential

Does your paper address subitem 21-ii?

Copy and paste relevant sections from the manuscript (include quotes in quotation marks "like this" to indicate direct quotes from your manuscript), or elaborate on this item by providing additional information not in the ms, or briefly explain why the item is not applicable/relevant for your study

This issue has been discussed widely from the counseling and mHealth system point of view. For example: "The counseling procedure of the main arm of the MySteps trial was implemented as intended regardless of the COVID-19 -induced challenges to patients with T2D. One reason may be that the procedure mostly included telephone contacts. Only a few sessions or contacts were missed which suggests that the number of sessions and contacts was feasible to the participants. The mean lengths of the individual sessions and contacts seemed also reasonable in clinical practice, although there was a large variation. The large variation probably illustrates the demand for adjusting the length of the sessions and contacts according to individual needs, which may not always be executable in real-life healthcare settings. The findings on fidelity and length of the sessions are in line with the most comparable study to the present one, where 83% of the patients (63% with T2D and 37% with COPD) received the intended intervention of four sessions and where most of the sessions exceeded the intended 20 minutes [65]."

OTHER INFORMATION

23) Registration number and name of trial registry

Does your paper address CONSORT subitem 23? *

Copy and paste relevant sections from the manuscript (include quotes in quotation marks "like this" to indicate direct quotes from your manuscript), or elaborate on this item by providing additional information not in the ms, or briefly explain why the item is not applicable/relevant for your study

ClinicalTrials.gov NCT04587414

24) Where the full trial protocol can be accessed, if available

Does your paper address CONSORT subitem 24? *

Cite a Multimedia Appendix, other reference, or copy and paste relevant sections from the manuscript (include quotes in quotation marks "like this" to indicate direct quotes from your manuscript), or elaborate on this item by providing additional information not in the ms, or briefly explain why the item is not applicable/relevant for your study

The full protocol is not separately available.

25) Sources of funding and other support (such as supply of drugs), role of funders

Does your paper address CONSORT subitem 25? *

Copy and paste relevant sections from the manuscript (include quotes in quotation marks "like this" to indicate direct quotes from your manuscript), or elaborate on this item by providing additional information not in the ms, or briefly explain why the item is not applicable/relevant for your study

"We thank all the patients with T2D participating in the study and the two study physiotherapists in Siun sote implementing the mHealth and counseling procedures of the study in a clinical setting. We are also grateful to the Ministry of Education and Culture, the Social Insurance Institution, and Research Committee of the Kuopio University Hospital Catchment Area for the State Research Funding for financing the study."

X27) Conflicts of Interest (not a CONSORT item)

X27-i) State the relation of the study team towards the system being evaluated

In addition to the usual declaration of interests (financial or otherwise), also state the relation of the study team towards the system being evaluated, i.e., state if the authors/evaluators are distinct from or identical with the developers/sponsors of the intervention.

	1	2	3	4	5	
subitem not at all important	○	○	○	○	○	essential

Does your paper address subitem X27-i?

Copy and paste relevant sections from the manuscript (include quotes in quotation marks "like this" to indicate direct quotes from your manuscript), or elaborate on this item by providing additional information not in the ms, or briefly explain why the item is not applicable/relevant for your study

"Conflicts of Interest: None declared."

About the CONSORT EHEALTH checklist

As a result of using this checklist, did you make changes in your manuscript? *

- ○ yes, major changes
- ○ yes, minor changes
- ◉ no

What were the most important changes you made as a result of using this checklist?

Oma vastauksesi

How much time did you spend on going through the checklist INCLUDING making changes in your manuscript *

I spent at least three hours.

As a result of using this checklist, do you think your manuscript has improved? *

- ○ yes
- ○ no
- ◉ Muu: I did not make any changes since the revisions suggested by the Editor

Would you like to become involved in the CONSORT EHEALTH group?

This would involve for example becoming involved in participating in a workshop and writing an "Explanation and Elaboration" document

- ○ yes
- ◉ no
- ○ Muu:

Tyhjennä valinta

Any other comments or questions on CONSORT EHEALTH

Oma vastauksesi

STOP - Save this form as PDF before you click submit

To generate a record that you filled in this form, we recommend to generate a PDF of this page (on a Mac, simply select "print" and then select "print as PDF") before you submit it.

When you submit your (revised) paper to JMIR, please upload the PDF as supplementary file.

Don't worry if some text in the textboxes is cut off, as we still have the complete information in our database. Thank you!

Final step: Click submit !

Click submit so we have your answers in our database!

Lähetä   Tyhjennä lomake

Älä koskaan lähetä salasanaa Google Formsin kautta.

Google ei ole luonut tai hyväksynyt tätä sisältöä. - Käyttöehdot - Tietosuojakäytäntö

Näyttääkö lomake epäilyttävältä? Ilmoita
